# Histone Modification Complex JMJ704‐HDA709 Negatively Regulates Salinity Tolerance in Rice

**DOI:** 10.1002/advs.75873

**Published:** 2026-06-01

**Authors:** Jing Wang, Zhengting Chen, Pengxiang Bai, Biao Liu, Lingling Meng, Jian Zhang, Xu Li, Dao‐Xiu Zhou, Xuan Ma, Yu Zhao

**Affiliations:** ^1^ National Key Laboratory of Crop Genetic Improvement Hubei Hongshan Laboratory Huazhong Agricultural University Wuhan China; ^2^ College of Agriculture Ningxia University Yinchuan China; ^3^ Institute of Plant Science Paris‐Saclay (IPS2) CNRS INRAE University Paris–Saclay Orsay France; ^4^ State Key Laboratory of Rice Biology and Breeding China National Rice Research Institute Hangzhou China

**Keywords:** JMJ704, HDA709, histone modification complex, salt stress, OsWRKY72

## Abstract

Soil salinity poses a significant threat to global crop productivity. Understanding the molecular mechanisms underlying plant response to salt stress is crucial for enhancing crop resilience and yield. Here, we show that histone demethylase JMJ704 interacts with HDA709, a deacetylase that is specific for H3K9ac, to synergistically modulate salt tolerance in rice. Genetic analysis revealed that overexpressing either *JMJ704* or *HDA709* enhanced salt sensitivity, whereas the *hda709* mutant exhibited a salt‐tolerant phenotype. Interestingly, while the *jmj704* mutant showed no significant change in salt sensitivity, the *jmj704hda709* double mutant demonstrated markedly improved salt tolerance. Immunoblot analysis showed bidirectional crosstalk between JMJ704‐catalyzed H3K4me3 demethylation and HDA709‐mediated H3K9ac deacetylation, with both enzymes exhibiting synergistic functional interplay. We further demonstrated that under salt stress, JMJ704 forms a chromatin‐modifying complex with HDA709, which, together with the transcription factor OsWRKY72, co‐regulates peroxidase and salt‐responsive genes. This recruitment facilitates transcriptional silencing through concurrent erasure of H3K4me3 and H3K9ac marks. Collectively, our work reveals that the JMJ704‐HDA709 complex interacts with OsWRKY72 to negatively regulate rice salt tolerance by modulating ROS metabolism and salt‐responsive genes. This finding provides evidence for the pivotal role of epigenetic regulation of plant abiotic stress responses and offers new insights for developing stress‐resilient crops.

## Graphical abstract

This study reveals that the rice histone demethylase JMJ704 interacts with HDA709―a H3K9ac deacetylase characterized herein―to form a chromatin‐modifying complex. Under salt stress, OsWRKY72 recruits this complex through interaction with JMJ704 to target loci, repressing the expression of oxidative stress and salt‐responsive genes via removal of H3K4me3 and H3K9ac, thereby negatively regulating rice salt tolerance. This epigenetic module provides key targets for developing stress‐resilient crops.

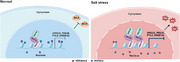

## 1 Introduction

Soil salinization has emerged as a critical global challenge threatening agricultural production and food security. The majority of staple crops exhibit a high degree of sensitivity to salinity, with elevated soil salinity levels significantly impeding crop growth and yield [1]. To cope with salt stress, plants have evolved a range of sophisticated physiological and molecular strategies, including maintaining ion homeostasis by restricting Na^+^ absorption and transport, as well as sequestering Na^+^ within vacuole [2, 3, 4, 5]. Concurrently, plants regulate their osmotic potential by synthesizing compatible osmolytes, such as proline, glycine‐betaine, trehalose, and other molecules, which stabilize cellular structures under stress [5, 6]. Furthermore, a robust antioxidant defense system is activated to scavenge reactive oxygen species (ROS) [7, 8]. Upon exposure to high salinity, plants swiftly initiate intricate signal transduction cascades, facilitating the transcription of downstream genes [9, 10]. These genetic activation, in turn, triggers stress defense mechanisms that fine‐tune the physiological and metabolic pathways, enabling the plants to adapt effectively to such harsh conditions [1, 5, 11].

Epigenetic modifications, such as histone acetylation and methylation, play a crucial role in responding to salt stress in plants by modulating the expression of salt‐responsive genes [12, 13, 14, 15]. In Arabidopsis, the RPD3‐like histone deacetylase HDA9 attenuates sensitivity to salinity by modulating histone acetylation levels at stress‐responsive loci [16]. Similarly, HDA19 compromises salt tolerance by decreasing histone acetylation levels at stress tolerance‐related genes and ABA receptor genes. Conversely, the histone deacetylases (HDACs) HDA5/14/15/18 augment salt resistance [17, 18]. In rice (*Oryza sativa*), histone deacetylase HDA710 suppresses salt tolerance by downregulating *LATE EMBRYOGENESIS ABUNDANT 3* (*OsLEA3*), *OsABI5*, *bZIP72*, and *Na^+^/H^+^ ANTIPORTER 1* (*NHX1*) through H4K5/K16 acetylation [19]. While HDA706 promotes stress resilience by inhibiting the negative regulator phosphatase 2C protein OsPP2C49 [20]. In addition to HDACs, histone methylation enzymes are also involved in regulating salt tolerance in plants. In particular, histone demethylase JMJ15 enhances salt resistance by removing the activating marks H3K4me3 from the promoters and coding regions of *WRKY46* and *WRKY70*, thereby suppressing their transcription in Arabidopsis [21]. In contrast, the rice histone methyltransferase SDG721 activates the expression of Na^+^/K^+^ transporter *OsHKT1;5* gene by depositing H3K4me3 at its promoter, thereby enhancing salt tolerance [22]. However, whether and how rice histone demethylases participate in regulating the salt stress response is unclear. Furthermore, little is known about the crosstalk between histone methylation and acetylation in modulating plant responses to abiotic stress.

Jumonji C (JmjC) domain‐containing proteins are recognized as conserved histone lysine demethylase across animals, plants, and fungi [23]. These JmjC proteins preferentially catalyze the removal of di‐ and tri‐methylations from histone lysine resides through ferrous ion (Fe[II]) and α‐ketoglutaric acid‐dependent oxidative reactions. The rice genome encodes 20 JmjC domain‐containing histone demethylases (named JMJ701‐JMJ720), which are classified into five distinct groups: JARID/KDM5, JMJD2/KDM4, JHDM2/KDM3, JMJD6, and JmjC domain‐only groups [24, 25]. Among these, members of the JARID group, including JMJ703 and JMJ704, have been identified as H3K4me3 demethylases [24, 26, 27]. JMJ703 affects plant stem elongation and panicle size by repressing the expression of cytokinin oxidase genes in rice [24, 28]. Recent study further indicated that it controls grain size and weight by regulating the grain size gene *GW2* and *OsLAC* [29]. Additionally, JMJ703 has been reported to play a negative role in drought tolerance in rice [30]. In contrast, the functional characterization of JMJ704, a homolog of JMJ703, remains relatively limited. It was recently shown that JMJ704 contributes to rice resistance against *Xanthomonas oryzae pv. Oryzae* (*Xoo*) by modulating H3K4me3 levels at negative regulators of disease resistance [26]. Nevertheless, whether JMJ704 is involved in salt stress response in rice remains unclear.

Salinity stress triggers an excessive accumulation of reactive oxygen species (ROS) within plant cells, resulting in lipid peroxidation of cell membrane, oxidation of biomolecules, and cytotoxicity [7, 31, 32]. Under salt‐alkali stress conditions, the timely activation of antioxidant defense mechanisms to scavenge ROS is critical for enhancing plant stress tolerance [31, 32]. Recently, a key gene, *AT1*, was identified to negatively regulate alkali tolerance in sorghum. This gene encodes the Gγ subunit, which modulates intra‐ and extracellular H_2_O_2_ levels through inhibiting the phosphorylation of the aquaporin PIP2;1 [33, 34]. Intriguingly, suppression of *GS3*, the rice ortholog of *AT1*, significantly enhances rice salt‐alkali tolerance and grain yield [33, 34]. These findings highlight the importance of maintaining ROS homeostasis for improving salt tolerance in plants. However, it is unclear whether epigenetic modifications are involved in regulating the response to salt stress by tuning antioxidant systems or ROS metabolism in plants.

In this work, we elucidated the interplay between the rice histone demethylase JMJ704 and HDA709, a H3K9ac deacetylase characterized herein, in modulating plant salt tolerance. Genetic analyses demonstrated that both JMJ704 and HDA709 function as negative regulators of salt tolerance in rice. Mechanistically, JMJ704‐HDA709 complex interacts with the transcription factor OsWRKY72 at peroxidase genes (*PRX22* and *PRX26*) and salt‐responsive genes, where it catalyzes the removal of H3K4me3 and H3K9ac marks to epigenetically repress their expression. Our results suggest that OsWRKY72, JMJ704, and HDA709 form a regulatory module to negatively regulate salt tolerance by suppression of ROS metabolism and salt‐responsive genes expression. Collectively, the findings not only deepen our understanding of the complex regulatory networks governing salt stress responses in rice but also provide the rationale for the development of novel epigenetic‐based strategies to enhance salt tolerance in crops.

## 2 Results

### 2.1 JMJ704 Negatively Regulates Rice Salt Tolerance

**FIGURE 1:**
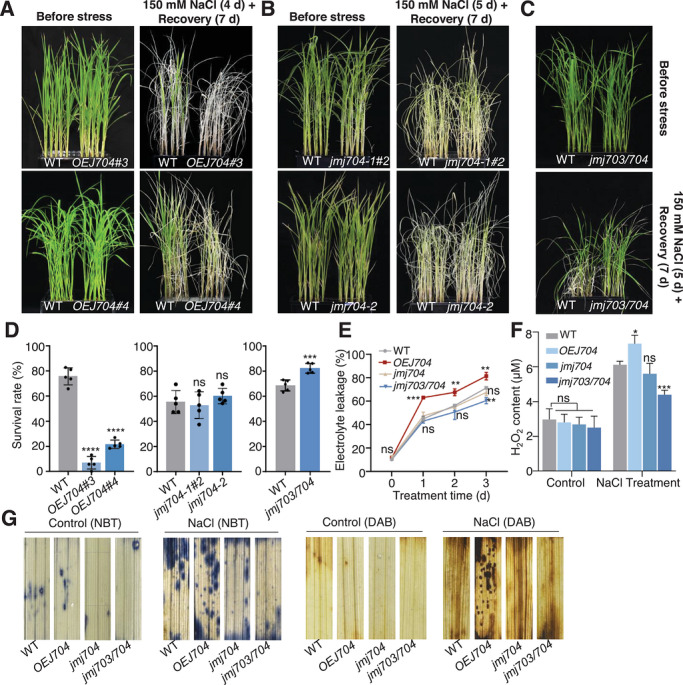
Effects of mutation and overexpression of *JMJ704* on rice tolerance to salinity stress. (A) Phenotypes of *JMJ704* overexpression lines (*OEJ704#3* and *OEJ704#4*), (B) *jmj704* mutants (*jmj704‐1#2*, *jmj704‐2*), and (C) *jmj703 jmj704* double mutants (*jmj703/704*) and wild‐type (WT) rice seedlings before salt treatment and recovered for 7 days after salt treatment (150 mM NaCl) for 4–5 days. (D) Survival rates of *OE‐JMJ704*, *jmj704*, *jmj703/704* double mutants and WT seedlings under salt stress treatment. Data are means ± SD (*n* = 5). (E) Electrolyte leakage rates of *OE‐JMJ704*, *jmj704*, *jmj703/704* and WT seedlings under salt treatment for different time points. Data are means ± SD (*n* = 3). (F) H_2_O_2_ contents of WT, *OE‐JMJ704, jmj704* and *jmj703/704* mutants under normal condition and salt stress (150 mm NaCl, for 24 h). Data are means ± SD (*n* = 3). Significances were tested by two‐tailed unpaired Student's *t*‐test (ns, not significant; ^*^, *P* < 0.05; ^**^, *P* < 0.01; ^***^, *P* < 0.001; ^****^, *P* < 0.0001). (G) DAB and NBT staining showing the ROS contents in leaves of WT, *OE‐JMJ704*, *jmj704* and *jmj703/704* mutants under normal condition and salt stress (150 mm NaCl, for 24 h). The *jmj704* refers to the *jmj704‐1#2* mutant line in panels E‐G.

To elucidate the function of histone demethylase JMJ704, we generated a *jmj704* mutant (*jmj704‐1*) using the CRISPR/cas9‐mediated gene editing system and two lines of *JMJ704* overexpression plants (*OEJ704#3* and *OEJ704#4*) (Figure S1A–C). Under normal condition, no visible developmental defects were observed in either *jmj704‐1* or *OE‐JMJ704* plants (Figure S1D,E). Immunoblotting analysis revealed that JMJ704 is a histone demethylase specific for H3K4me3 in rice (Figure S2A,B), consistent with previous study [26]. Furthermore, *JMJ704* was highly expressed in rice roots (Figure S2C), and its transcript levels were significantly induced by high salinity (150 mM NaCl), osmotic stress (20% PEG6000), and heat stress (42°C) (Figure S2D–F). To assess whether JMJ704 is involved in the plant response to salt stress, we evaluated the salt tolerance of *OE*‐*JMJ704* plants and the *jmj704‐1* mutant at the seedling stage. Compared to wild‐type (WT) plants, *OE‐JMJ704* plants displayed a pronounced salt‐sensitive phenotype when subjected to 150 mm NaCl treatment (Figure 1A). Conversely, the *jmj704‐1* mutant showed no sensitivity to salt stress (Figure 1B, upper panel). To further validate these observations, we obtained an additional loss‐of‐function mutant of *JMJ704* (renamed *jmj704‐2*) that harbored a T‐DNA insertion in the fourth intron of the *JMJ704* genomic DNA in Zhonghua11 (ZH11) background (Figure S1A). The *jmj704‐2* mutant exhibited a similar phenotype to *jmj704‐1* under salt stress (Figure 1B, lower panel). The survival rate of *OE‐JMJ704* plants was significantly reduced, while the survival rate of *jmj704* mutants was comparable to that of WT plants under salt stress (Figure 1D).

To investigate the role of JMJ704 in rice salt tolerance, we evaluated the germination and growth of *OE‐JMJ704*, *jmj704*, and WT plants on ½ MS medium supplemented with different concentrations of NaCl (0 [control], 50, 100, and 150 mm). The results showed that *OE‐JMJ704* plants were hypersensitive to salinity, with their root and shoot growth notably inhibited under 50 mm NaCl treatment. In contrast, the roots of the *jmj704* mutant were significantly longer than those of the WT under 100 mm or 150 mm NaCl treatment (Figure S3), suggesting a slight improvement in salt tolerance for the mutant, although no obvious difference from WT plants was observed in the aboveground part under salt stress (Figure 1B,D). The rice histone demethylase JMJ703 is homologous to JMJ704, both of which are specific for H3K4me3. To study whether JMJ703 is involved in plant response to salinity, we analyzed physiological phenotypes using *JMJ703* overexpression lines (*OEJ703#1* and *OEJ703#2*) and two CRISPR/cas9‐editing mutant lines (*jmj703#1* and *jmj703#2*; Figure S4A). Under salt stress, the phenotypes of *OE‐JMJ703* plants and *jmj703* mutants were similar to those of *OE‐JMJ704* and *jmj704* mutants: *OE‐JMJ703* plants exhibited high sensitivity to salinity, while *jmj703* mutants showed no clear difference compared to the WT (Figure S4B,C). These results suggest that JMJ704 may function redundantly with JMJ703 in the salt stress response. Therefore, we generated a *jmj703/704* double mutant using the CRISPR/cas9 system and evaluated its salt tolerance. Interestingly, the *jmj703/704* plants displayed robust salt tolerance under 150 mm NaCl treatment (Figure 1C), with a significantly higher survival rate than WT plants under salt stress (Figure 1D).

To further explore the physiological mechanisms of *JMJ704* in plant responses to salinity, we measured the electrolyte leakage rate in *OE‐JMJ704*, *jmj704*, *jmj703/704*, and WT plants under salt stress. Electrolyte leakage, which is related to K^+^ efflux from plant cells mediated by plasma membrane cation conductance under abiotic stress [35], was significantly increased in *OE‐JMJ704* plants compared to WT during salt treatment, while a significant decrease in electrolyte leakage was observed in *jmj703/704* mutants after 3 days of salt treatment (Figure 1E). This indicated that the membrane permeability of *OE‐JMJ704* plants was severely affected by salt stress. Additionally, we detected the ROS levels using diaminobenzidine (DAB) and nitrotetrazolium blue chloride (NBT) staining and measured H_2_O_2_ levels in *OE‐JMJ704*, *jmj704*, *jmj703/704*, and WT under salt stress. Compared to WT plants, *OE‐JMJ704* plants displayed a significant increase in H_2_O_2_ levels, while *jmj703/704* mutants exhibited a large decrease of H_2_O_2_. No clear differences in H_2_O_2_ levels were observed between *jmj704* and WT plants (Figure 1F). The results from DAB and NBT staining were consistent with the H_2_O_2_ levels (Figure 1G), indicating that salt stress induced excessive ROS production in *OE‐JMJ704* plants, causing severe oxidative damage. Together, these results demonstrated that JMJ704 negatively regulates salt tolerance in rice.

### 2.2 JMJ704 is Involved in the Transcripts of ROS Metabolism and Salt‐responsive Genes

**FIGURE 2:**
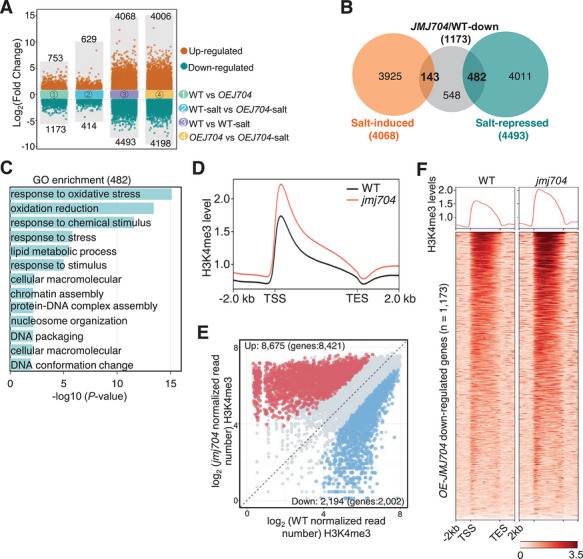
JMJ704‐mediated H3K4me3 demethylation represses oxidative stress‐related and salt‐responsive genes in rice. (A) Volcano plots showing the differentially expressed genes (DEGs) identified between *OE‐JMJ704* and WT seedlings under normal condition and salt stress. (B) Venn diagram showing the salt‐responsive genes downregulated in *OE‐JMJ704* plants. (C) GO enrichment analysis of the 482 salt‐repressed genes that are downregulated in *OE‐JMJ704* plants. (D) Metaplot showing the H3K4me3 modification profiles of the *jmj704* and WT seedlings. (E) Scatter plot showing the differential modification peaks of H3K4me3 in *jmj704* compared to WT. The *x*‐axis and *y*‐axis represent normalized read counts at each peak in the WT and *jmj704*, respectively. Blue dots indicate peaks with reduced H3K4me3 in *jmj704*; red dots indicate peaks with increased H3K4me3. Differential peaks (fold change > 2, *P* < 0.01) identified in both replicates were considered significantly changed. (F) Metaplots and heatmaps showing the H3K4me3 levels in WT and *jmj704* mutant for the downregulated genes in *OE‐JMJ704* plants.

To dissect the target genes regulated by JMJ704 under salt stress, we performed RNA sequencing (RNA‐seq) on 2‐week‐old seedlings of *OE‐JMJ704* and WT plants, both subjected to 12 h of salt treatment. Each genotype was analyzed in triplicate under salt stress or normal condition (Figure S5A; Table S1). Principal component analysis (PCA) revealed distinct transcriptome differences between *OE‐JMJ704* and WT under normal condition, with substantial transcriptome alterations observed in both *OE‐JMJ704* and WT before and after salt stress treatment (Figure S5B). Differentially expressed genes (DEGs) were identified (log2 |fold change|≥ 1, false discovery rate [FDR] < 0.05) between *OE‐JMJ704* plant and WT under normal or salt stress condition. There were 753 up‐regulated and 1173 down‐regulated genes identified in *OE‐JMJ704* compared to WT under normal condition (Figure 2A). Gene Ontology (GO) enrichment analysis indicated that the down‐regulated genes were predominantly involved in oxidation‐reduction processes, oxidative stress response, and secondary metabolic processes (Figure S6A), suggesting that genes related to redox homeostasis were suppressed in *OE‐JMJ704* plants. Under salt stress condition, a comparable number of up‐ and down‐regulated genes were identified in both *OE‐JMJ704* and WT (Figure 2A), with over 60% of the DEGs overlapping between the two genotypes (Figure S6B). Interestingly, almost half (41%, 482 genes) of the genes downregulated in the *OE‐JMJ704* under normal condition (*OEJ704* vs. WT) were also repressed by salt stress in WT plants (Figure 2B). These genes were mainly enriched in response to oxidative stress, oxidation‐reduction, and response to stress stimulus (Figure 2C). Notably, among these genes were 23 peroxidase (PRX) encoding genes and several previously reported important salt‐responsive genes, such as *OsASR1, OsABCG5, OsPYL6, OsRCAR10*, and K^+^ transporter OsHAK1, and the low‐affinity cation transporter OsLCT1 (Figure 2C; Figure S5C; Dataset S1). The transcriptomic analysis suggested that JMJ704 is required for transcriptional repression under salt stress in rice.

To study the chromatin function of JMJ704 in rice, we performed chromatin immunoprecipitation followed by high‐throughput sequencing (ChIP‐seq) with an anti‐H3K4me3 antibody using 2‐week‐old seedlings of the *jmj704* mutant and WT plants (Figure S7A, Table S2). The ChIP‐seq analysis detected approximately 22 260 and 24 756 H3K4me3 peaks in WT and the *jmj704* mutant, respectively. Metaplots showed a marked increase in overall H3K4me3 levels across genic regions in the *jmj704* mutant compared to WT (Figure 2D). Differential analysis identified 8675 peaks (8421 genes) with significantly increased (fold change > 2, *P* < 0.01), and 2194 peaks (2002 genes) with decreased H3K4me3 levels in the mutant (Figure 2E). Genes with elevated H3K4me3 were primarily enriched in molecular metabolic process, RNA metabolic processing, and transferase activity (Figure S7B). By integrating RNA‐seq data from *OE‐JMJ704* plants, metaplots analysis revealed that the downregulated genes in *OE‐JMJ704* exhibited higher H3K4me3 levels in *jmj704* mutants than WT (Figure 2F), suggesting that JMJ704‐mediated removal of H3K4me3 transcriptionally represses these target genes.

### 2.3 JMJ704 Interacts with Histone Deacetylase HDA709

**FIGURE 3:**
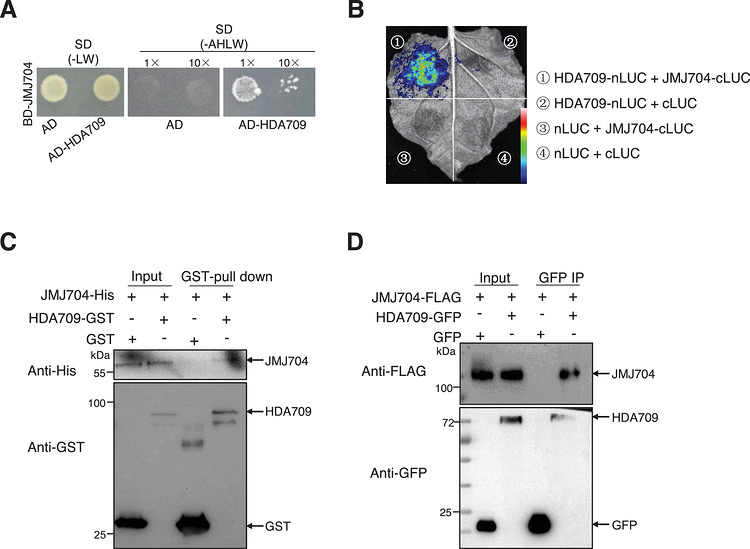
JMJ704 interacts with histone deacetylase HDA709 in vitro and in vivo. (A) Yeast two‐hybrid (Y2H) assay showing the interaction between JMJ704 and HDA709. (B) Split luciferase complementation assay of JMJ704 and HDA709 in tobacco (*N. benthamiana*). Constructs encoding nLUC‐tagged HDA709 or nLUC alone were co‐infiltrated into tobacco leaves with cLUC‐tagged JMJ704 or cLUC alone, as indicated in white font. Infiltrated leaves were harvested and dark‐adapted for 5 min before luminescence detection. (C) Pull‐down assay of JMJ704 and HDA709. JMJ704‐6 × His was incubated with GST or HDA709‐GST immobilized on GST beads, and JMJ704 was pulled down from the HDA709‐GST conjugated GST beads. (D) Co‐immunoprecipitation assay of JMJ704 and HDA709 in tobacco leaves. *35Spro:JMJ704‐FLAG* construct was transiently transfected into tobacco leaves alone or co‐transfected with *35Spro:HDA709‐GFP*.

To investigate the mechanism underlying JMJ704 regulates the salt tolerance in rice, we conducted yeast two‐hybrid (Y2H) assays using JMJ704 as a bait to identify its potential interaction proteins. Intriguingly, HDA709 (LOC_Os11g09370), a histone deacetylase belonging to the RPD3/HDA1 subfamily, was found to be an interacting partner of JMJ704 (Figure 3A). In contrast, no interaction was detected between JMJ703 and HDA709. Furthermore, a total of 18 transcription factors (TFs) were found to interact with JMJ704 (Table S3). We further validated the interactions between JMJ704 and HDA709 by split‐luciferase complementation assays with HDA709‐nLUC and JMJ704‐cLUC in tobacco (*Nicotiana benthamiana*) cells (Figure 3B). Furthermore, in vitro pull‐down assays using recombinant HDA709 tagged with glutathione S‐transferase (GST) and JMJ704 tagged with 6 × His‐tag expressed in *Escherichia coli* (Figure 3C), and co‐immunoprecipitation by transient expression of HDA709‐GFP and JMJ704‐FLAG proteins in tobacco leaves further confirmed their association (Figure 3D). Deletion analysis via Y2H revealed that the JmjN and JmjC domains of JMJ704, together with the HDAC domain of HDA709, are indispensable for their mutual interaction (Figure S8).

### 2.4 HDA709 is a H3K9 Deacetylase in Rice

**FIGURE 4:**
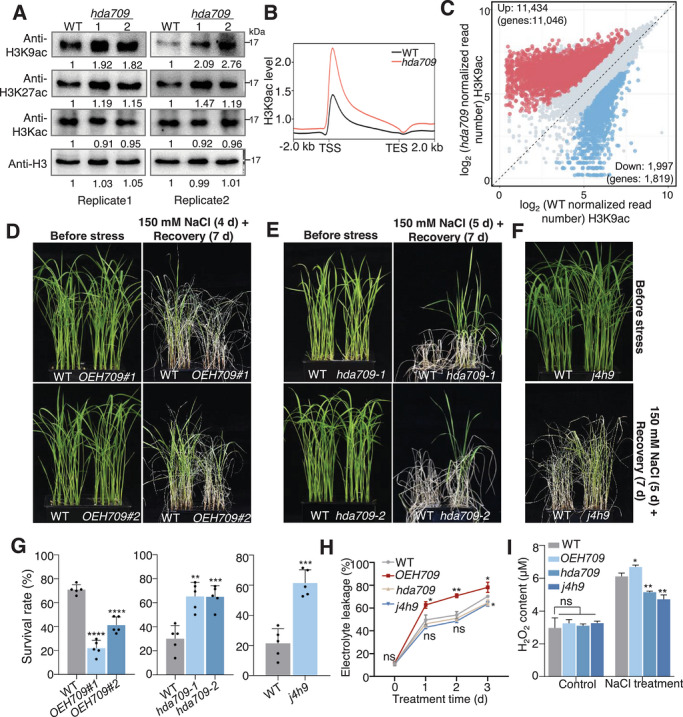
Histone deacetylase HDA709 negatively regulates salt tolerance in rice. (A) Immunoblotting assay showing that HDA709 is a deacetylase specific to H3K9ac and H3K27ac in rice. The immunoblot signals were quantified by ImageJ. (B) Metaplot showing the H3K9ac modification profiles of the *hda709* and WT. (C) Scatter plot showing the H3K9ac differential peaks identified in *hda709* relative to WT. The x‐axis and y‐axis represent normalized read counts at each peak in the WT and *hda709*, respectively. Blue dots indicate peaks with reduced H3K9ac in *hda709*; red dots indicate peaks with increased H3K9ac. Differential peaks (fold change > 2, *P* < 0.01) identified in both replicates were considered significantly changed. (D–F) Phenotype of the *HDA709* overexpression plants, *hda709* mutant, *hda709* and *jmj704* double mutants (*j4h9*), and WT seedlings before salt treatment and recovered for 7 days after salt treatment (150 mm NaCl) for 5 days. (G) Survival rates of *OE‐HDA709*, *hda709*, *jmj704hda709 (j4h9)* double mutants and WT seedlings under salt stress treatment. Data are means ± SD (*n* = 5). (H) Electrolyte leakage rates of *OE‐HDA709*, *hda709*, *jmj704hda709 (j4h9)*, and WT seedlings under salt treatment at different time points. Data are means ± SD (*n* = 3). (I) H_2_O_2_ contents of WT, *OE‐HDA709*, *hda709*, and *jmj704hda709* (*j4h9*) mutants under normal condition and salt stress (150 mm NaCl, for 24 h). Data are means ± SD (*n* = 3). Significances were calculated by two‐tailed unpaired Student's *t*‐test (ns, not significant; ^*^, *P* < 0.05; ^**^, *P* < 0.01; ^***^, *P* < 0.001; ^****^, *P* < 0.0001).

Subcellular localization of HDA709 in rice protoplast demonstrated its presence within the rice nucleus (Figure S9A, upper panel). The subcellular localization was subsequently confirmed by anti‐FLAG immunostaining analysis in rice root tip cells (Figure S9A, lower panel). Expression profile analysis showed that *HDA709* was highly expressed in ovary and roots (Figure S9B). To investigate the function of HDA709, we generated two *HDA709* overexpressing lines (*OEH709#1* and *OEH709#2*) and CRISPR/cas9‐mediated knockout *hda709* mutants (*hda709‐1*, *hda709‐2*) (Figure S10A–C). Immunoblotting assays revealed that HDA709 deacetylated H3K9ac and H3K27ac in transgenic plants (Figure 4A; Figure S9D). To confirm this, we performed ChIP‐seq with an anti‐H3K9ac antibody on 2‐week‐old seedlings of *hda709* mutants and WT plants (Figure S7A). The ChIP‐seq analysis detected about 21 049 H3K9ac peaks in WT seedlings. Metaplot analysis revealed a genome‐wide elevation of H3K9ac levels in the *hda709* mutant compared to WT (Figure 4B). Specifically, 11 434 peaks (11 046 genes) exhibited a significant increase (fold change > 2, *P* < 0.01) in H3K9ac, while only 1997 peaks (1819 genes) showed a decrease in the mutant (Figure 4C). Functional enrichment analysis indicated that genes with hyperacetylated H3K9ac were predominantly involved in nucleotide binding, gene expression, RNA metabolic process, and hydrolase activity (Figure S7C). Collectively, these results demonstrated that HDA709 deacetylates H3K9ac mark in rice.

### 2.5 HDA709 Participates in Modulating Salt Stress Response in Rice

The expression of *HDA709* was significantly induced by salt stress (Figure S9C). To evaluate whether HDA709 is involved in salt stress response, 2‐week‐old seedlings of *hda709* mutants and WT plants were subjected to 150 mm NaCl for 5 days, followed by a 7‐day recovery. Notably, *hda709* mutants displayed a salt‐tolerant phenotype under salt stress treatment compared to WT, whereas the *OE‐HDA709* plants were hypersensitive to the stress (Figure 4D,E). Furthermore, *hda709* mutants had higher survival rates and lower electrolyte leakage ratios under stress. By contrast, *OE‐HDA709* plants significantly reduced survival rates and increased electrolyte leakage ratios (Figure 4G,H). Detection of ROS levels under salt stress showed that H_2_O_2_ content in *hda709* was significantly lower than in WT, whereas *OE‐HDA709* accumulated higher H_2_O_2_ levels (Figure 4I). DAB and NBT staining also revealed similar results to H_2_O_2_ levels observed in *HDA709* transgenic plants (Figure S10D). These results indicated that, similar to JMJ703/704, HDA709 also plays a negative role in salt stress response in rice.

**FIGURE 5:**
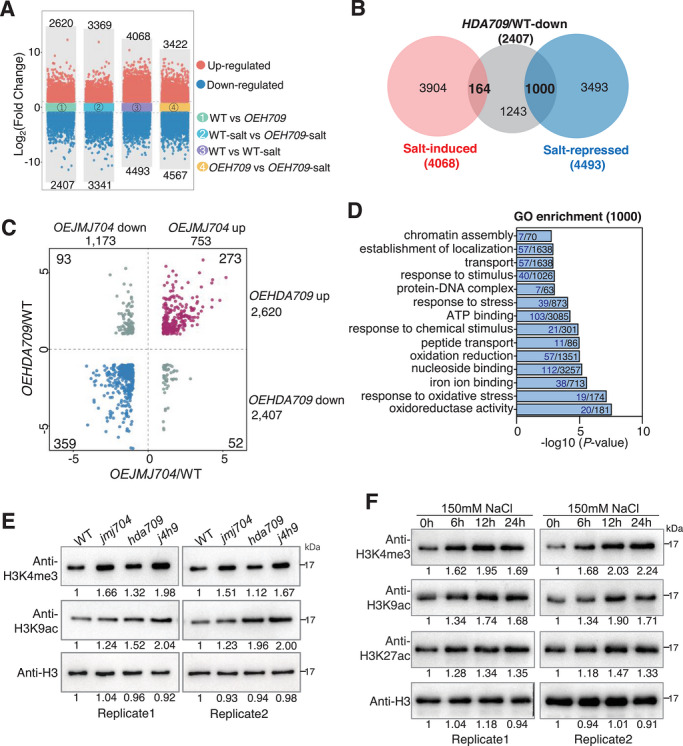
HDA709 and JMJ704 co‐repress salt‐responsive genes through modulating H3K9ac and H3K4me3 levels in rice. (A) Volcano plots of DEGs identified between *OE‐HDA709* and WT seedlings under salt stress treatment. (B) A Venn diagram showing the salt‐responsive genes in WT are downregulated in *OE‐HDA709* plants. (C) Scatter plot of gene expression changes (fold change > 2, FDR < 0.05) in *OE‐JMJ704* and *OE‐HDA709* compared with the WT. (D) GO enrichment analysis of the 1000 salt‐repressed genes that are downregulated in *OE‐HDA709* plants. (E) Immunoblot analysis of the H3K4me3 and H3K9ac levels in *jmj704, hda709*, and *jmj704hda709* (*j4h9*) double mutants. Two replicates are shown. The corresponding quantitative analysis is presented in Figure S11A. (F) Dynamic changes of histone modifications during time‐course salt stress. Two replicates are shown. The immunoblot signals were quantified using ImageJ. The corresponding quantitative analysis is presented in Figure S11B.

We further performed RNA‐seq analysis using 2‐week‐old seedlings of *OE‐HDA709* and WT plants, both of which were subjected to 12 h salt treatment (Figure S5A). Identification of DEGs (log2FC > 1, FDR < 0.05) revealed that 2620 genes were upregulated and 2407 were downregulated in *OE‐HDA709* compared to WT under normal condition (Figure 5A). Notably, a large proportion of the downregulated genes in *OE‐HDA709* (1000 genes, accounting for 41.5%) overlapped with those repressed by salt stress in WT (Figure 5B). These overlapping genes were primarily enriched in response to oxidative stress (Figure 5D), which is highly consistent with the transcriptome of *OE‐JMJ704*, with a core set of 273 co‐upregulated and 359 co‐downregulated genes in both OE transgenic lines compared to WT (Figure 5C). This shared gene expression profile strongly suggests that HDA709 and JMJ704 target a similar gene set to regulate salt tolerance in rice.

### 2.6 JMJ704 and HDA709 Synergistically Regulate the Salt Tolerance of Rice

The direct interaction between HDA709 and JMJ704 (Figure 3) and their similar effects on plant response to salt suggest that they might synergistically cooperate in stress response. To test the hypothesis, we generated a *jmj704hda709* double mutant by crossing *jmj704‐1* and *hda709‐1 and* evaluated its salt tolerance. Under 150 mm NaCl treatment for 5 days, followed by a 7‐day recovery, the survival rate of the *jmj704hda709* mutant was significantly higher than that of WT plants (Figure 4F,G). Additionally, the H_2_O_2_ and ROS levels in *jmj704hda709* were significantly lower than those in WT plants (Figure 4I; Figure S10D), indicating a high salt tolerance of the double mutant.

To examine the dynamics of chromatin modification in rice during salt stress treatment, we performed immunoblotting assays and found that the levels of H3K4me3, H3K9ac, and H3K27ac in WT plants were significantly induced by salt treatment (Figure 5F, Figure S11B), consistent with previous reports in Arabidopsis [36]. H3K4me3 and H3K9ac are well‐known chromatin markers for active genes, and their modification levels are positively correlated with gene expression levels. In addition, both types of modifications are positively correlated [37]. To analyze whether these two enzymes mutually affect their histone modification activities, we performed immunoblot analyses to test H3K4me3 levels in *hda709* mutants and H3K9ac levels in *jmj704* mutants. Interestingly, we found that H3K4me3 levels in *hda709* and H3K9ac levels in *jmj704* were increased, respectively (Figure 5E, Figure S11A,C,D). Furthermore, the levels of H3K4me3 and H3K9ac in the *jmj704hda709* mutants were remarkably increased compared to WT, *jmj704*, or *hda709* mutants (Figure 5E, Figure S11A). Comparative analysis of ChIP‐seq data from *jmj704* and *hda709* mutants revealed approximately 7295 genes that exhibited concurrent gains of H3K4me3 and H3K9ac in these two mutants (Figure S11E). Functional enrichment analysis revealed that these genes were mainly involved in molecular metabolic processes, transferase activity, and RNA metabolic processes (Figure S11F). Together, these results suggest a genome‐wide crosstalk between H3K4me3 and H3K9ac modifications likely controlled synergistically by JMJ704 and HDA709.

### 2.7 JMJ704 Interacts with Transcription Factor OsWRKY72

**FIGURE 6:**
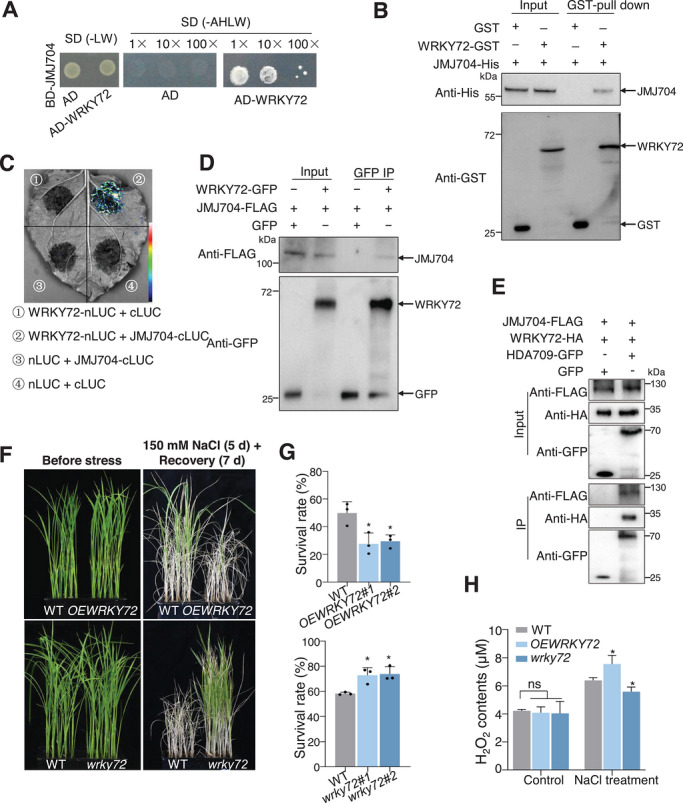
JMJ704 interacts with WRKY72 in vitro and in vivo. (A) Y2H assay demonstrating the interaction between JMJ704 and WRKY72. (B) Pull‐down assay of the interaction of JMJ704 and WRKY72. JMJ704‐6 × His was incubated with GST or WRKY72‐GST in GST beads, and JMJ704 was pulled down from the WRKY72‐GST conjugated GST beads. (C) In vivo split luciferase complementation assays of the interaction between JMJ704 and WRKY72 in tobacco leaves. nLUC‐tagged WRKY72 or nLUC alone were co‐infiltrated into tobacco leaves with cLUC‐tagged JMJ704 or cLUC alone, as indicated in white font. Infiltrated leaves were harvested and dark‐adapted for 5 min before luminescence detection. (D) In vivo co‐immunoprecipitation assay confirming the interaction between JMJ704 and WRKY72 in tobacco leaves. The *35Spro:JMJ704‐FLAG* construct was transiently transfected into tobacco leaves alone or co‐transfected with *35Spro:WRKY72‐GFP*. (E) Co‐immunoprecipitation assays demonstrating the in vivo formation of the JMJ704‐HDA709‐OsWRKY72 complex. The *35Spro:JMJ704‐FLAG* and *35Spro:WRKY72‐HA* constructs were transiently co‐expressed in tobacco leaves with either *35Spro:HDA709‐GFP* or GFP alone. Immunoprecipitation was performed using anti‐GFP beads. Protein levels in the Input and IP samples were detected using anti‐FLAG, anti‐HA, and anti‐GFP antibodies. (F) Phenotypes of *OE‐WRKY72, wrky72*, and WT seedlings before salt treatment and recovered for 7 days after salt treatment (150 mm NaCl) for 5 days. (G) Survival rates of *OE*‐*WRKY72* and *wrky72* mutant seedlings under salt stress treatment (*n* = 3). (H) H_2_O_2_ contents of WT, *OE*‐*WRKY72*, and *wrky72* under normal condition and salt stress (150 mm NaCl, for 24 h). Data are means ± SD (*n* = 3). Significances were tested by Student's two‐tailed unpaired *t*‐test (ns, not significant; ^*^, *P* < 0.05).

The above data demonstrated that both JMJ704 and HDA709 negatively regulate salt tolerance in rice. To elucidate the mechanism by which the histone modifiers target specific genes, from the above‐mentioned Y2H screening, we found that JMJ704 could interact with multiple WRKY transcription factors (Table S3), including OsWRKY72 (Figure 6A), a transcription factor recently reported to play a role in modulating salt tolerance in rice [38]. To confirm the interaction, we performed in vitro pull‐down assays with recombinant OsWRKY72 tagged with GST and JMJ704 tagged with a 6 × His tag, and split‐luciferase complementation imaging assays in tobacco cells and co‐immunoprecipitation in transfected tobacco leaves, all tests indicated that JMJ704 physically interacted with OsWRKY72 (Figure 6B–D). Given that JMJ704 interacts with both OsWRKY72 and HDA709, we hypothesized that these three proteins might form a ternary complex to exert their functions collectively. To test this hypothesis, we co‐expressed JMJ704‐FLAG, OsWRKY72‐HA, and HDA709‐GFP in tobacco leaves and subsequently performed co‐immunoprecipitation assays using anti‐GFP beads to pull down the protein complex (Figure 6E). The results confirmed the formation of a ternary protein complex consisting of JMJ704, OsWRKY72, and HDA709.

### 2.8 OsWRKY72 Interacts with JMJ704‐HDA709 Complex to Suppress the ROS Metabolism and Salt‐responsive Genes

Recent studies have reported that allelic variations of *OsWRKY72* in two rice cultivars confer high salt tolerance to their hybrid F1 [38]. We found that *OsWRKY72* was prominently expressed in rice roots and significantly induced by salt stress (Figure S12A,B). To further dissect the functional mechanisms of this TF in response to salt stress, we evaluated the salt tolerance of *OsWRKY72* overexpression plants (*OE*‐*WRKY72*) and *wrky72* mutant produced previously [39]. Under 150 mm NaCl treatment for 5 days the *wrky72* mutant showed a strong salt‐tolerant phenotype compared to WT. Specifically, the survival rate of *wrky72* was significantly increased and the H_2_O_2_ content was markedly reduced under salt stress compared to WT plants (Figure 6F–H, Figure S12C). In contrast, the *OE*‐*WRKY72* plants exhibited a salt‐sensitive phenotype with a significantly decreased survival rate and increased H_2_O_2_ levels under salt stress (Figure 6F–H, Figure S12C). These observations demonstrated that OsWRKY72 negatively regulates salt tolerance in rice, in agreement with the recent study.

**FIGURE 7:**
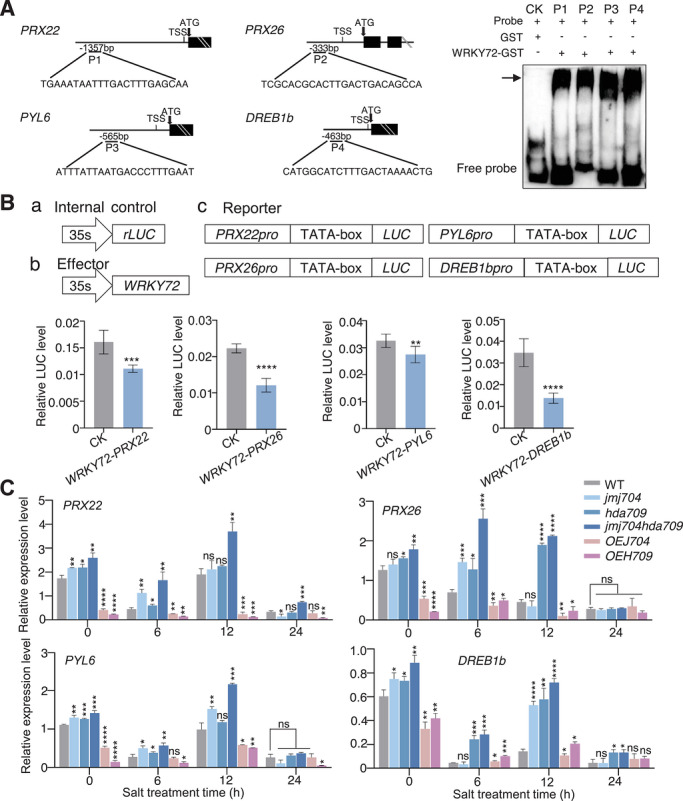
OsWRKY72 represses its target genes by interacting with JMJ704 to remove H3K4me3 at their promoters. (A) Electrophoretic mobility shift assays (EMSA) showing that OsWRKY72 directly binds to the (T)TGAC(C/T) motif in the promoters of *PRX22*, *PRX26*, *PYL6*, and *DREB1b*. Left, DNA sequence of the probes used in EMSA. Right, arrow indicates the OsWRKY72 and biotin‐labeled DNA complex. (B) Dual‐luciferase assay showing that OsWRKY72 represses *PRX22*, *PRX26*, *PYL6*, and *DREB1b* expression in rice protoplasts. (C) Relative expression levels of *PRX22, PRX26, PYL6*, and *DREB1b* in *OE‐JMJ704* (*OEJ704*)*, jmj704* mutant, *OE*‐*HDA709* (*OEH709*), *hda709* mutant, *jmj704hda709* double mutants and WT plants under salt stress treatment for different time points (150 mm NaCl treatment for 0 [control], 6, 12, 24 h). Data are means ± SD (*n* = 3). Significances were calculated by two‐tailed unpaired Student's *t*‐test (ns, not significant; ^*^, *P* < 0.05; ^**^, *P* < 0.01; ^***^, *P* < 0.001; ^****^, *P* < 0.0001).

To study how OsWRKY72 regulated the expression of downstream target genes, we conducted an in‐depth analysis of DNA affinity purification sequencing (DAP‐seq) data for OsWRKY72 [40], and combined the DEGs identified from the transcriptomes of *OE‐JMJ704* and *wrky72* [40] plants. In total, 100 DEGs were identified as common target genes for both OsWRKY72 and JMJ704 (Figure S12D, Dataset S2). GO analysis showed that these genes are predominantly enriched in oxidation‐reduction, response to oxidative stress, and response to stress/stimulus (Figure S12E). Among these genes, we found 2 *OsAATs*, 10 *PRX* genes, as well as some genes/TFs previously reported to be involved in salinity and/or drought responsive, such as ABA receptor *Pyrabactin Resistance 1‐like 6* (*PYL6*), *Root Specific Pathogenesis‐related protein 10* (*RSOsPR10*), *Chilling Inducible 1* (*OsChI1*), *Dehydration‐Responsive Element‐Binding protein 1b* (*DREB1b*), *MYB8*, *MYB30*, *NAC104*, and *OsWRKY7* (Dataset S2). To identify direct targets from these candidate genes, we screened for the presence of W‐box motif (WRKY protein binding motif) in their promoters. Four representative genes (*OsPRX22*, *OsPRX26*, *OsPYL6*, and *OsDREB1b*) were selected to test OsWRKY72 binding in electrophoretic mobility shift assay (EMSA). The results demonstrated that OsWRKY72 could directly bind to the promoters of *OsPRX22*, *OsPRX26*, *OsPYL6*, and *OsDREB1b* (Figure 7A; Figure S13A–D). Moreover, we observed that salt stress markedly induced OsWRKY72 protein accumulation and target DNA‐binding (Figure S14A,B). Transient expression assays revealed that OsWRKY72 significantly repressed the transcription of the tested target genes (*OsPRX22*, *OsPRX26*, *OsPYL6*, and *OsDREB1b*) (Figure 7B).

**FIGURE 8:**
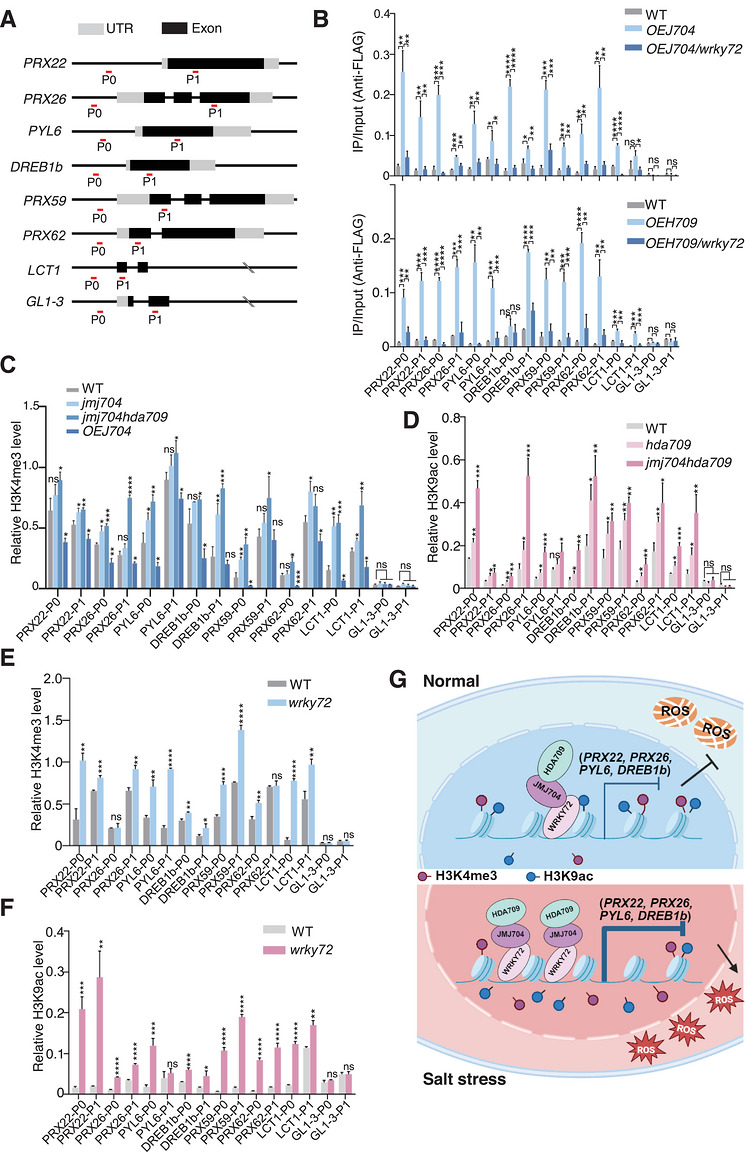
OsWRKY72 interacts with JMJ704‐HDA709 complex to epigenetically regulate salt‐responsive genes and salt tolerance in rice. (A) Schematic diagrams of the promoter and genic regions of *PRX22*, *PRX26*, *PYL6*, *DREB1b*, *PRX59*, *PRX62*, and *LCT1*. Red horizontal lines indicate the regions analyzed by ChIP‐qPCR with antibodies against FLAG, H3K4me3, H3K9ac. *GL1‐3* as a negative control. (B) ChIP‐qPCR detection of JMJ704 and HDA709 occupancy at the target genes (*PRX22*, *PRX26*, *PYL6*, *DREB1b, PRX59, PRX62*, and *LCT1*) in *ubi:JMJ704‐FLAG* and *ubi:HDA709‐FLAG* in the *wrky72* mutant background (*OEJ704/wrky72* and *OEH709/wrky72*) using an anti‐FLAG antibody. (C) ChIP‐qPCR detection of H3K4me3 levels of the target genes (*PRX22, PRX26, PYL6, DREB1b, PRX59, PRX62*, and *LCT1*) in *jmj704, jmj704hda709*, *OE‐JMJ704* and WT plants grown under normal condition. (D) ChIP‐qPCR detection of H3K9ac modification levels at the target genes in *hda709, jmj704hda709* and WT plants grown under normal condition. (E‐F) ChIP‐qPCR detection of H3K4me3 (E) and H3K9ac (F) modification levels at the target genes in the *wrky72* mutant. Data are means ± SD (*n* = 3). Significances were calculated by two‐tailed unpaired Student's *t*‐test (ns, not significant; ^*^, *P* < 0.05; ^**^, *P* < 0.01; ^***^, *P* < 0.001; ^****^, *P* < 0.0001). (G) Proposed model of OsWRKY72 interacting with the JMJ704‐HDA709 complex to epigenetically regulate rice salt tolerance. Under normal condition (upper panel), the transcription of *PRX22*, *PRX26*, *PYL6*, and *DREB1b* is not suppressed, and these genes maintain the homeostasis of ROS metabolism and are involved in regulating the growth and development in rice plants; under salt stress condition (lower panel), OsWRKY72 binds to the promoters of *PRX22*, *PRX26*, *PYL6*, and *DREB1b*, interacting with histone demethylase JMJ704 to remove H3K4me3 modifications in the promoters. JMJ704 interacts with the histone deacetylase HDA709 to form a transcriptional repression complex, which removes H3K4me3 and H3K9ac modifications to more efficiently suppress downstream gene expression. The repression of *PRX* genes in turn causes an imbalance in ROS metabolism and promotes salt sensitivity in rice.

RT‐qPCR showed that the expression levels of these target genes were significantly higher in the *jmj704*, *hda709*, and *jmj704hda709* mutants than *OE‐JMJ704*, *OE‐HDA709*, and WT plants. Additionally, the transcription of these genes was significantly repressed by salt treatment in WT, consistent with RNA‐seq data, while the transcript levels of these genes were significantly increased or unchanged in *jmj704, hda709*, and *jmj704hda709* mutants but dramatically decreased in *OE*‐*JMJ704* and *OE‐HDA709* during salt treatment compared to WT (Figure 7C; Figure S15). Anti‐FLAG ChIP‐qPCR analysis of *OE*‐*JMJ704* and *OE‐HDA709* plants confirmed that JMJ704 and HDA709 indeed target these genes (Figure 8A,B). Analysis of three additional candidate target genes (*OsPRX59*, *OsPRX62*, and *OsLCT1*), which contain the W‐box motif, revealed that JMJ704 and HDA709 can also target these genes (Figure 8A,B). Furthermore, H3K4me3 levels at the promoter and genic region of these genes (*OsPRX22*, *OsPRX26*, *OsPRX59*, *OsPRX62*, *OsPYL6*, *OsLCT1*, and *OsDREB1b*) were significantly decreased in *OE‐JMJ704* but increased in *jmj704* mutant plants under salt stress (Figure 8C). Similarly, H3K9ac levels of these genes were significantly increased in *hda709* mutant under salt stress (Figure 8D). Notably, H3K4me3 and H3K9ac levels of these target genes were both prominently enhanced in *jmj704hda709* double mutant (Figure 8C,D). This indicated that JMJ704 and HDA709 targeted to OsWRKY72‐binding genes and removed the H3K4me3 and H3K9ac to suppress their expression under salt stress. To investigate whether the JMJ704‐ and HDA709‐mediated histone modifications of these target genes depend on OsWRKY72, we tested H3K4me3 and H3K9ac levels in *wrky72* mutants by ChIP‐qPCR. The results revealed that the enrichment of H3K4me3 and H3K9ac at the promoter or genic regions of these target genes (*OsPRX22, OsPRX26, OsPRX59, OsPRX62, OsPYL6, OsDREB1b*, and *OsLCT1*) was significantly increased in *wrky72* mutants compared to WT (Figure 8E,F). Consistently, ChIP‐seq data demonstrated that the overall levels of H3K4me3 and H3K9ac in the OsWRKY72 target genes were significantly elevated in *jmj704* and *hda709* mutants (Figure S16A). To further confirm whether the recruitment of JMJ704 and HDA709 to these target genes is mediated by OsWRKY72, we generated *ubi:JMJ704‐FLAG* and *ubi:HDA709‐FLAG* constructs in the *wrky72* mutant background and assessed their binding enrichment by ChIP‐qPCR using an anti‐FLAG antibody. Notably, the abundance of both JMJ704 and HDA709 at these target loci was significantly reduced in the *wrky72* mutant background compared to the wild‐type background (*OE‐JMJ704* and *OE‐HDA709*, Figure 8B). Together, these findings demonstrate that the targeting of JMJ704 and HDA709 to downstream genes is dependent on OsWRKY72 under salt stress.

To further examine the potential influence of histone modification levels on the activity of encoded proteins or enzymes, the activity of peroxidase (POD, *PRX* encoding enzymes) was detected in *JMJ704* and *HDA709* overexpression and mutant plants. The results showed that the activity of POD was significantly elevated in *hda709* but decreased in *OE‐JMJ704* and *OE‐HDA709* plants under salt stress (Figure S16C). Similar results were also observed in *OE‐WRKY72* and *wrky72* mutant plants (Figure S16C). Collectively, these results demonstrated that OsWRKY72 negatively regulated rice salt tolerance by interacting with JMJ704‐HDA709 complex to epigenetically repress ROS scavenging and salt‐responsive genes.

## 3 Discussion

Chromatin modifications play a crucial role in facilitating plant adaptation to environmental cues by regulating gene transcription [12, 14]. Understanding the mechanisms through which chromatin‐modifying factors regulate plant responses to abiotic stresses is of great importance for improving crop resilience. In this study, we elucidate the mechanism by which the histone modification complex JMJ704‐HDA709 synergistically regulates salt stress response in rice. Specifically, we showed that salt stress significantly induces both the transcription levels and protein accumulation of OsWRKY72, promoting its binding to downstream target genes, including peroxidase genes and salt‐responsive genes. OsWRKY72 represses the target genes, likely by interacting with the chromatin‐modifying complex JMJ704‐HDA709 to remove H3K4me3 and H3K9ac from the target genes, thereby negatively modulating salt tolerance in rice (Figure 8G). Furthermore, this work provides evidence that crosstalk between histone modifications plays a critical role in orchestrating plant responses to abiotic stress.

It has been postulated that crosstalk of histone modifications with similar functions can reinforce the robustness of a chromatin state [41]. The mechanisms underlying this crosstalk are diverse, usually with one modification potentially catalyzing the occurrence of another [42]. It has been shown that histone demethylases/deacetylases can interact to control gene expression and are involved in various biological processes in plants [43, 44]. For instance, RPD3/HDA1 class I histone deacetylase HDA6 interacts with the Lysine‐Specific Demethylase 1 (LSD1)‐like histone demethylases LDL1 and LDL2 and co‐regulates the expression of *TOC1* (*TIMING OF CAB EXPRESSION 1*), a central oscillator in the circadian clock through histone demethylation and deacetylation [8]. Additionally, HDA6 interacts with the LSD1‐type histone demethylase FLD to modulate flowering time in Arabidopsis [44]. In this work, we show that histone demethylase JMJ704 interacts with HDA709 to cooperatively modulate salt tolerance in rice. Under salt stress condition, the *hda709* mutant exhibited a salt‐tolerant phenotype, while the *jmj704* mutant maintained wildtype‐like aboveground phenotype but exhibited root salt tolerance (Figure S3), and the *jmj704hda709* plants displayed an enhanced salt‐tolerant phenotype (Figure 4F). Increases of H3K4me3 in *hda709* mutant and H3K9ac in *jmj704* mutant (Figure S11C,D) suggest that these two histone modification enzymes function as a mutually dependent entity, reciprocally reinforcing each other. Therefore, the crosstalk between these two modifications or the interactions between JMJ704 and HDA709 can enable rice to rapidly respond to salinity stress and swiftly activate or suppress the related genes expression under stress condition. In plants, several histone‐modifying enzymes interact to coordinately regulate biological processes, typically exhibiting sequential patterns in the deposition and removal of histone modifications at specific gene loci. For instance, in Arabidopsis, histone deacetylase HDA9 and Polycomb Repressive Complex 2 (PRC2) function in concert to mediate H3K27 deacetylation followed by trimethylation at the *FLOWERING LOCUS C* (*FLC*) locus, resulting in *FLC* repression and playing a crucial role in regulating the developmental transition to flowering [45]. Recent studies in rice have demonstrated that GA‐induced transcriptional activation is associated with a rapid accumulation of H3K9ac mediated by HDA702, which is subsequently followed by the removal of H3K27me3 [46]. In contrast to these sequential modification patterns, our study demonstrated that the interaction between JMJ704 and HDA709 likely mediates the simultaneous removal of activation marks from the downstream genes, while exhibiting a mutually reinforcing regulatory relationship between these two enzymes.

In the present study, the *jmj704* mutant exhibited a weak salt‐tolerant phenotype in roots (Figure S3), whereas no obvious differences were observed in shoots compared to the WT under salt condition. This may be attributed to functional redundancy between JMJ704 and JMJ703, both of which function as H3K4me3‐specific demethylases. Indeed, the *jmj703jmj704* double mutant exhibited a clear salt‐tolerant phenotype under salt treatment (Figure 1C). *JMJ704* was predominantly expressed in rice roots, while *JMJ703* showed high expression in roots, stems, and pistils (Figure S2C; Figure S4D), suggesting both overlapping and distinct functional roles. Recent studies have revealed that these two genes function redundantly in regulating rice heading data. Specifically, they interact with each other and associate with the PRC2 subunits to form a transcriptional repression complex that suppresses *OsLFL1* expression, thereby promoting flowering in rice [47]. In addition, JMJ703 has been reported to influence plant height by modulating cytokinin‐mediated stem elongation [24]. In contrast, the *jmj704* mutant exhibits no obvious developmental defects. However, JMJ704 has been shown to participate in the resistance response to bacterial blight in rice [26]. These findings suggest that JMJ704 and JMJ703 share partial functional redundancy in regulating rice development and stress responses, while also exhibiting functional divergence: JMJ703 appears to be preferentially involved in developmental regulation, whereas JMJ704 plays a more prominent role in abiotic and biotic stress responses. Such functional differences are likely attributable to variations in their downstream target genes. In the present work, we found that the target genes regulated by JMJ704 are primarily enriched in multiple metabolic pathways and redox metabolism.

Cellular ROS metabolism is closely related to plant development and stress responses [8, 32]. ROS homeostasis in plant cells can profoundly impact plant resilience under stress condition [7, 31]. In this study, both *OE‐JMJ704* and *OE*‐*HDA709* plants exhibited increased sensitivity to salinity. Physiologically, ROS (H_2_O_2_ and O^2−^) levels were significantly enhanced in these OE lines under salt stress condition, whereas they were reduced in the *jmj704* and *hda709* mutants (Figure 1F,G; Figure 4I; Figure S10D). This indicated that JMJ704 and HDA709 were involved in regulating rice salt tolerance by modulating ROS metabolism. Transcriptomics analyses further demonstrated that many redox‐related genes were downregulated in the *OE‐JMJ704* plants (Figure 2B, C; Figure S5C), confirming that the expression of *JMJ704* directly affects ROS metabolism. Moreover, the transcription factor OsWRKY72 regulates rice salt tolerance by affecting ROS scavenging. Recent studies have shown that allelic variations in *OsWRKY72* between the paternal and maternal lines of the hybrid rice Chaoyou1000 result in differential repression of downstream genes *OsGSTU26* and *OsAAT30*, thus conferring the hybrid F1 with higher ROS scavenging ability and salt tolerance than its parents [38]. Here, we discovered that OsWRKY72 directly binds to the promoters of multiple class III peroxidase genes (*PRX22*, *PRX26*). Class III *PRX* genes encode heme‐containing enzymes that oxidize phenolic compounds while consuming H_2_O_2_ and/or generating other forms of ROS, such as OH ^–^ or O^2 –^ [48]. PRX enzymatic activity is triggered by ROS produced in response to various biotic/abiotic stresses [49]. Under salt stress condition, POD activity was significantly reduced in *OE‐WRKY72*, *OE‐JMJ704*, and *OE‐HDA709* plants, whereas a marked increase was observed in their corresponding mutant lines, except for *jmj704*, which showed no significant difference (Figure S16C). This enzymatic activity was highly consistent with the fluctuations in H_2_O_2_ content. These observations provide compelling evidence that OsWRKY72 negatively regulates rice salt tolerance by recruiting the JMJ704‐HDA709 chromatin‐modifying complex, which epigenetically suppresses the transcription of *PRX* genes. This transcriptional repression compromises ROS scavenging capacity in plant cells under salt stress condition, thereby enhancing salt sensitivity. The epigenetic mechanism by which OsWRKY72 negatively regulates salt tolerance in rice may serve as a brake on stress defense, playing a critical role in balancing plant growth and stress responses. Recent studies have revealed that OsWRKY72 plays a crucial role in regulating leaf angle, grain size, and yield in rice [40, 50], highlighting its importance as a multifunctional transcription factor. OsWRKY72 is thus involved not only in growth, development, and yield but also in regulating both biotic and abiotic stress resistance in rice [26, 38, 40, 50]. Our work elucidates the epigenetic mechanism by which OsWRKY72 negatively regulates salt tolerance, offering valuable insights for the fine‐tuning of its expression to enhance stress resistance and yield potential in rice.

Collectively, our findings establish that the OsWRKY72‐JMJ704‐HDA709 regulatory module functions as a critical molecular switch in the negative regulation of salt tolerance in rice. This mechanistic discovery not only advances our fundamental understanding of epigenetic regulation in plant stress adaptation but also provides a new strategy for developing stress‐resilient crops through targeted genetic manipulation of epigenetic regulatory networks.

## 4 Materials and Methods

### 4.1 Plant Materials and Abiotic Stresses Treatments

Rice (*Oryza sativa* ssp. *Japonica*) variety Zhonghua11 (ZH11) was used to produce the *JMJ704* and *HDA709* transgenic plants in this study. The *jmj704‐1* and *hda709* mutants were generated using a CRISPR/Cas9 system [51], and the sgRNA sequences were designed using CRISPR‐P2.0 (http://crispr.hzau.edu.cn/CRISPR2/) (Table S4). Genotypes of the mutants were decoded by DSDecode software [52]. The *jmj704‐2* mutant is a previously characterized T‐DNA insertion line in the ZH11 background [26]. The *JMJ704* and *HDA709* overexpression lines were generated by transforming *Ubipro:JMJ704‐3×FLAG* and *Ubipro:HDA709‐3×FLAG* constructs into ZH11, respectively. The *Ubipro:JMJ704‐3×FLAG* and *Ubipro:HDA709‐3×FLAG* constructs were produced by inserting the CDS regions of *JMJ704* and *HDA709* into a pCAMBIA1301‐3×FLAG vector respectively, which contains a maize *Ubiquitin* promoter. The *OsWRKY72* mutants and overexpression lines, were described previously [39]. The double‐mutants of *jmj704* and *hda709* were created by crossing the *jmj704‐1* and *hda709‐1* mutant plants. Tobacco (*N. benthamiana*) plants used for transient expression were grown in soil for 6 weeks at 20 ± 2°C, with an 8 h photoperiod.

For rice seedlings’ abiotic stresses treatments, seeds were first immersed in water at 37°C for 2 days, then placed the germinated seeds into a bottomless 96‐well plate and grown in hydroponic nutrient solution for 2 weeks at 25°C with a 14 h light/10 h dark cycle. Various stress treatments were applied to seedlings at early developmental stages. For heat stress, 14‐day‐old seedlings were incubated at 42°C in a growth chamber for 5 to 7 days. The control group was maintained at 25°C. For salt and osmotic stress, NaCl (150 mm) and PEG6000 (20%) were added to the nutrient solution for 4 to 5 days, respectively. After the stress treatments, seedlings were returned to normal condition for 7 days. Survival rates were then determined by calculating the ratio of viable seedlings to the total number of seedlings using a formula: (live plants/total plants) × 100%, the determination was performed with three biological replicates, and each replicate with 40 plants. For the abiotic stress treatments involving overexpression lines, the negative lines segregated from the first generation of the overexpression plants were used as controls.

### 4.2 Measurement of Electrolyte Leakage Rate

The electrolyte leakage measurement was conducted following a previously described method with minor modifications [53]. Two fully expanded leaves from separate plants were cut into segments of uniform size and immersed in 8 mL of double‐distilled water in a 10 mL test tube. The samples were then incubated for 24 h at 25°C with continuous shaking at 100 rpm. Initial conductivity (R1) was measured using a conductivity meter (Model DDS‐IIA, China). Subsequently, the test tubes were placed in boiling water for 20 min and cooled naturally to room temperature, and the final conductivity (R2) was measured. The relative electrolyte leakage rate was calculated as the ratio of R1 to R2.

### 4.3 Determination of H_2_O_2_ Concentrations and POD Activity

Fourteen‐day‐old rice seedlings were treated with 150 mm NaCl for 24 h, while control seedlings were maintained under normal condition. For the H_2_O_2_ concentration assay, fresh leaf samples (approx. 0.1 g) from the salt treatment groups and the control group were respectively collected and ground in liquid nitrogen, and then homogenized in 10 mm K‐phosphate buffer (pH 6.5). The homogenate was centrifuged at 12 000 rpm at 4°C for 10 min. The supernatant was retained for measuring H_2_O_2_ content using an H_2_O_2_ Quantitative Assay Kit (Sangon Biotech, C500069‐0250). For the peroxidase (POD) activity assay, rice leaf samples (0.1 g) were ground in liquid nitrogen, homogenized with the extraction solution on ice, and then centrifuged at 8000 rpm at 4°C for 10 min. The supernatant was collected for measuring the POD activity using a Peroxidase Activity Assay Kit (Shanghai Enzyme‐linked Biotechnology, ML076330).

### 4.4 DAB and NBT Staining Assays

NBT and DAB staining were performed as previously described [22]. 14‐day‐old rice seedlings were treated with 150 mm NaCl for 24 h, while control plants were maintained under normal condition. For NBT staining, the leaves were collected and vacuum‐infiltrated for 30 min, and then incubated for 12 h at room temperature in a solution containing 0.05% NBT (*w*/*v*) and 10 mM NaN_3_ in 10 mm potassium phosphate buffer (pH 7.8). For DAB staining, leaves were vacuum‐infiltrated for 1 h and then incubated for 24 h in a solution containing 0.1% DAB tetrahydrochloride (*w*/*v*) in distilled water (pH 5.8). The reactions were terminated by transferring the leaves to 90% ethanol in a boiling water bath until the chlorophyll was completely removed. The stained leaves were photographed by a stereomicroscope (Nikon, SMZ25).

### 4.5 Subcellular Localization Assays

For subcellular localization assays, plasmids containing the *35Spro:GFP‐HDA709* fragment were introduced into rice protoplasts that were isolated from 14‐day‐old rice seedlings by a method described previously [54]. After incubation for 12 h, the protoplasts were imaged using a laser confocal microscope (Olympus, FV1200).

### 4.6 Yeast Two‐hybrid Assays

For yeast two‐hybrid assay, the full‐length CDS regions of *HDA709* and Os*WRKY72* were cloned into a pGADT7 vector, respectively, and the *JMJ704* CDS region was cloned into a pGBKT7 vector. The full‐length CDS and the truncated regions of *JMJ704* and *HDA709* were obtained by PCR using the specific primers (Table S4). The pGADT7 and pGBKT7 vectors were co‐transformed into a yeast (*Saccharomyces cerevisiae*) strain AH109. The transformed yeast cells were first incubated on SD/‐Trp/‐Leu medium at 28°C for 3 days. Subsequently, the clones were transferred to SD/‐Ade/‐His/‐Leu/‐Trp medium to assay protein‐protein interactions.

### 4.7 In Vitro Pull‐down Assays

For in vitro pull‐down assays, GST‐tagged HDA709, GST‐tagged WRKY72, and His‐tagged JMJ704 were constructed and expressed in *Escherichia coli* cells. The JMJ704‐6 × His construct contained only the JmjN and JmjC domains, representing a truncated form of JMJ704. This His‐tagged JMJ704 fragment was incubated with either GST alone, HDA709‐GST, or WRKY72‐GST, each immobilized on GST beads (ABclonal, AS044). The proteins were then pulled down and analyzed by immunoblotting using anti‐GST antibody (Abcam, Ab19256, 1:4,000 dilution) and anti‐His antibody (Abcam, Ab9108, 1:4000 dilution).

### 4.8 Split‐LUC Complementation Assays

The full‐length CDS regions of *HDA709* and Os*WRKY72* were cloned in‐frame upstream of the sequence encoding nLUC. The CDS region of *JMJ704* was cloned in‐frame downstream of the sequence encoding cLUC. Plasmids of different combinations were transiently co‐infiltrated into tobacco leaves. The designated pairs of transformed constructs were then infiltrated into the abaxial epidermis of 6‐week‐old tobacco leaves using a needleless syringe, with at least three tobacco plants used for each combination. After 48 h of transfection, the transfected leaves were treated with 1 mm luciferin (Gold Biotechnology) and kept in the dark for 10 min. Luciferase bioluminescence images were captured using the Chemi‐Image System (Tanon 5200Multi, China).

### 4.9 Co‐immunoprecipitation Assays

For the co‐immunoprecipitation assay, plasmids containing the constructs *35Spro:JMJ704‐FLAG*, *35Spro:HDA709‐GFP*, and *35Spro:WRKY72‐HA* or *35Spro:WRKY72‐GFP* were co‐transfected into tobacco leaves. After transformation for 72 h, the leaves were collected and lysed in co‐immunoprecipitation buffer (10 mm Tris‐HCl, pH 7.5, 150 mm NaCl, 0.5 mm EDTA, 1% [*v/v*] Triton X‐100, 1% [*w/v*] sodium deoxycholate, 0.1% [*w/v*] SDS, 1 mm DTT, 1 mm PMSF, and 15 U/mL DNase I) for 30 min, then centrifugation at 16 000 × g at 4°C for 5 min to remove cellular debris. The supernatant was transferred to a new tube and incubated with GFP‐conjugated beads (ABclonal, AE079) overnight. The precipitated proteins were then analyzed by immunoblotting with anti‐FLAG antibody (Sigma, F3165, 1:4000 dilution), anti‐GFP antibody (Abmart, M20004, 1:4000 dilution), and anti‐HA antibody (Abmart, M20003, 1:4000 dilution).

### 4.10 RNA‐seq and Data Analyses

Total RNA was isolated from 2‐week‐old rice seedlings of the following lines using TRIzol Reagent (Invitrogen, USA, Cat.15596026): wild type (WT), *jmj704‐1#2* mutant line (2‐bp deletion), *OEJMJ704#4* line, *hda709‐2* mutant (1‐bp insertion), and *OEHDA709#1* line. A total of 2 µg RNA per sample was used for mRNA isolation. RNA‐seq libraries were prepared using the TruSeq RNA Sample Preparation Kit (Illumina, USA), according to the manufacturer's protocol. The libraries were sequenced on an Illumina HiSeq 2500 instrument with paired‐end (150 bp) sequencing. The raw reads were filtered using Trimmomatic (version 0.36) to remove adapter and low‐quality reads. Clean reads were aligned to the reference genome (MSU 7.0, http://rice.plantbiology.msu.edu/) using Hisat2 (version 2.1.0) [55]. Gene expression levels were calculated by Cufflinks (version 2.2.1) with parameters for strand‐specific RNA‐seq. Differentially expressed genes (DEGs) were identified using Cuffdiff (v2.2.1) program [56], with thresholds set at an adjusted log_2_ |fold change| ≥ 1 and false discovery rate < 0.05.

### 4.11 Histone Extraction and Western Blot

An EpiQuik Total Histone Extraction Kit (Epigentek USA, OP‐0006‐100) was used to extract total histone‐enriched fractions from 14‐day‐old rice seedlings. The extracted histone proteins were analyzed using immunoblot analysis with the following antibodies: anti‐H3K4me3 (Abcam, ab8580, 1:1000 dilution), anti‐H3K9me2 (Abcam, ab1220, 1:1000 dilution), anti‐H3K27me3 (Millipore, 07–449, 1:1000 dilution), anti‐H3K36me3 (Abcam, ab9050, 1:1000 dilution), anti‐H3K9ac (Millipore, 07–352, 1:1000 dilution), anti‐H3K14ac (Abcam, ab52946, 1:1000 dilution), anti‐H3K23ac (Millipore, 07–355, 1:1000 dilution), anti‐H3K27ac (Abcam, ab4729, 1:1000 dilution), anti‐H3 (Abcam, ab1791, 1:1000 dilution), anti‐H3Kac (Millipore, 17–615, 1:1000 dilution), anti‐H4 (Abcam, ab177840, 1:1000 dilution), anti‐H4K5ac (Millipore, 07–327, 1:1000 dilution), anti‐H4K16ac (Millipore, 07–329, 1:1000 dilution), anti‐H4Kac (Millipore, 06–598, 1:1000 dilution), and peroxidase‐conjugated goat anti‐rabbit antibody (Abbkine, A21020, 1:10 000 dilution). Immunoblotting results were quantified using ImageJ (v1.6.0_24).

### 4.12 Reverse Transcription and RT‐qPCR

For first‐strand cDNA synthesis, 1 µg of total RNA was reverse‐transcribed in a 20 µL reaction volume using a Reverse Transcription Kit (Vazyme, R233‐01), according to the manufacturer's instructions. RT‐qPCR was performed with the following cycling conditions: 95°C for 10 s, 45 cycles of 95°C for 5 s, and 60°C for 40 s. Disassociation curve analysis was performed at 95°C for 15 s, 60°C for 20 s, and 95°C for 15 s. Three biological replicates were performed, each replicate with three technical repeats. Data were collected using the ABI PRISM 7500 sequence detection system. The rice *ACTIN1* gene was used as the internal control. The primers used for RT‐qPCR are listed in Table S4.

### 4.13 Electrophoretic Mobility Shift Assay

The OsWRKY72 protein fusion with GST tag was expressed in *Escherichia coli BL21* (DE3) cells and purified using GST agarose (ABclonal, AS044) following the manufacturer's instructions. For DNA probes, the Oligonucleotides of the target gene were synthesized and labeled with 5'‐biotin. The double‐stranded probes were obtained by mixing equal amounts of complementary single‐stranded oligonucleotides at 95°C for 2 min, followed by gradual cooling from 72°C to room temperature for annealing. The reactions were incubated at room temperature for 20 min and then resolved on a 6% (*w*/*v*) DNA retardation gel by electrophoresis at 100 V for 1–2 h and transferred to a nylon membrane. Biotin signals were detected using the Light Shift Chemiluminescent EMSA Kit (Thermo Scientific, 20148) according to the manufacturer's instructions. Primers used in this study are listed in Table S4.

### 4.14 Dual‐luciferase Transient Expression Assay

The promoter of the target gene (from −2000 to +100 bp relative to the ATG) was amplified from WT plants and used to drive reporter gene expression. The WRKY72 coding sequence driven by the 35S promoter was cloned into an effector vector. The effector, reporter, and a control plasmid were co‐transfected into rice protoplasts using PEG‐mediated transformation. Following transfection, protoplasts were incubated in the dark at 28°C for 16–18 h and then collected by centrifugation at 100 × g for 8 min. Luciferase activities were measured with the Dual‐Luciferase Reporter Assay Kit (E1910; Promega) using a microplate reader (Tecan, Mannedorf, Switzerland), and relative LUC/REN ratios were calculated. Data are presented as the mean of three technical replicates, and significant differences were determined by Student's t‐test. The experiment was independently repeated three times.

### 4.15 ChIP‐seq and Data Analysis

Chromatin immunoprecipitation (ChIP) was performed as previously described [57]. Briefly, 2 g of rice seedling leaves from the following lines were cross‐linked with 1% (v/v) formaldehyde: wild type (WT), *jmj704‐1#2* mutant line (2‐bp deletion), *OEJMJ704#4* line, *hda709‐2* mutant (1‐bp insertion), and *OEHDA709#1* line. Chromatin was extracted and sonicated to an average size of ∼200 bp. The fragmented chromatin was incubated overnight with antibody‐conjugated beads (Invitrogen, 10001D). After extensive washing, the immunoprecipitated chromatin was de‐cross‐linked and purified. The un‐immunoprecipitated chromatin served as the input control. Both immunoprecipitated and input DNA samples were analyzed by high‐throughput sequencing or RT‐qPCR using gene‐specific primers listed in Table S4. The following antibodies were used: anti‐H3K4me3 (Abcam, ab8580), anti‐H3K9ac (Millipore, 07–352), and anti‐FLAG (Sigma, F3165).

ChIP‐seq libraries were constructed from immunoprecipitated DNA and sequenced on an Illumina NovaSeq X Plus platform. Raw reads were processed with Trim Galore (v0.6.6) to remove adapters and low‐quality sequences. Clean reads were aligned to the rice reference genome (MSU7.0) using Bowtie2 (v2.4.1). MACS2 (v2.2.7.1) software was used to call histone modifications (H3K4me3 and H3K9ac) peaks. Differential peak analysis was conducted using DiffBind (v3.8.4) with the DESeq2 method, significant differential peaks were defined as those with *P* < 0.01 and |Fold change| > 2.

### 4.16 Accession Numbers

The RNA‐seq data produced in this study are deposited to NCBI Sequence Read Archive under accession number: PRJNA1224201 (http://www.ncbi.nlm.nih.gov/bioproject/1224201). The ChIP‐seq data produced in this study are deposited to China National Center for Bioinformation (CNCB) under accession number: PRJCA049593. Genes mentioned in this study can be retrieved from the Rice Genome Annotation Project website (http://rice.plantbiology. msu.edu/) using the following accession numbers: *JMJ704* (LOC_Os05g23670), *JMJ703* (LOC_Os05g10770), *HDA709* (LOC_Os11g09370), *WRKY72* (LOC_Os11g29870), *PRX22* (LOC_Os01g73200), *PRX26* (LOC_Os02g14170), *PYL6* (LOC_Os03g18600), *DREB1b* (LOC_Os09g35010), *PRX59* (LOC_Os04g59150), *PRX62* (LOC_Os04g59200), *LCT1* (LOC_Os06g38120).

### 4.17 Statistical Analysis

All statistical analyses and data visualization were performed using GraphPad Prism (version 8.3.0) and R (version 4.4.1). Data are presented as mean ± standard deviation (SD). Statistical significance between two groups was calculated using a two‐tailed unpaired Student's *t*‐test, and multiple comparisons were evaluated using one‐way ANOVA with Tukey's multiple comparisons test. A *P*‐value < 0.05 was considered statistically significant. Detailed statistical parameters, including sample sizes (*n*) and biological replicates, are provided in the figure legends or methods section.

## Author Contributions

J.W. performed most of the experimental work and data analysis; X.M. did bioinformatics analysis; Z.C., P.B., B.L., L.M., and X.L. participated in experimental work; J.Z. provided the *WRKY72* mutants and overexpression materials; D.‐X.Z. revised the manuscript; Y.Z. designed and supervised the work; Y.Z. and X.M. wrote and revised the paper with input from J.W.

## Conflicts of Interest

The authors declare no conflicts of interest.

## Supporting information




**Supporting File 1**: advs75873‐sup‐0001‐SuppMat.pdf.

**Supporting File 2**: advs75873‐sup‐0002‐Supplementary Table S1–S4.xlsx.

**Supporting File 3**: advs75873‐sup‐0003‐Data.zip.

## Data Availability

The data that support the findings of this study are openly available in NCBI Sequence Read Archive, CNCB at http://www.ncbi.nlm.nih.gov/bioproject/1224201, reference number PRJNA1224201, PRJCA049593.
