# Supplementary material for: Histone Modification Complex JMJ704‐HDA709 Negatively Regulates Salinity Tolerance in Rice
Source: Adv Sci (Weinh). 2026 Jun 1;13(47):e75873. doi: 10.1002/advs.75873 (PMC13335786; doi:10.1002/advs.75873)
Supplement: Supplementary file 1 [file ADVS-13-e75873-s003.pdf]

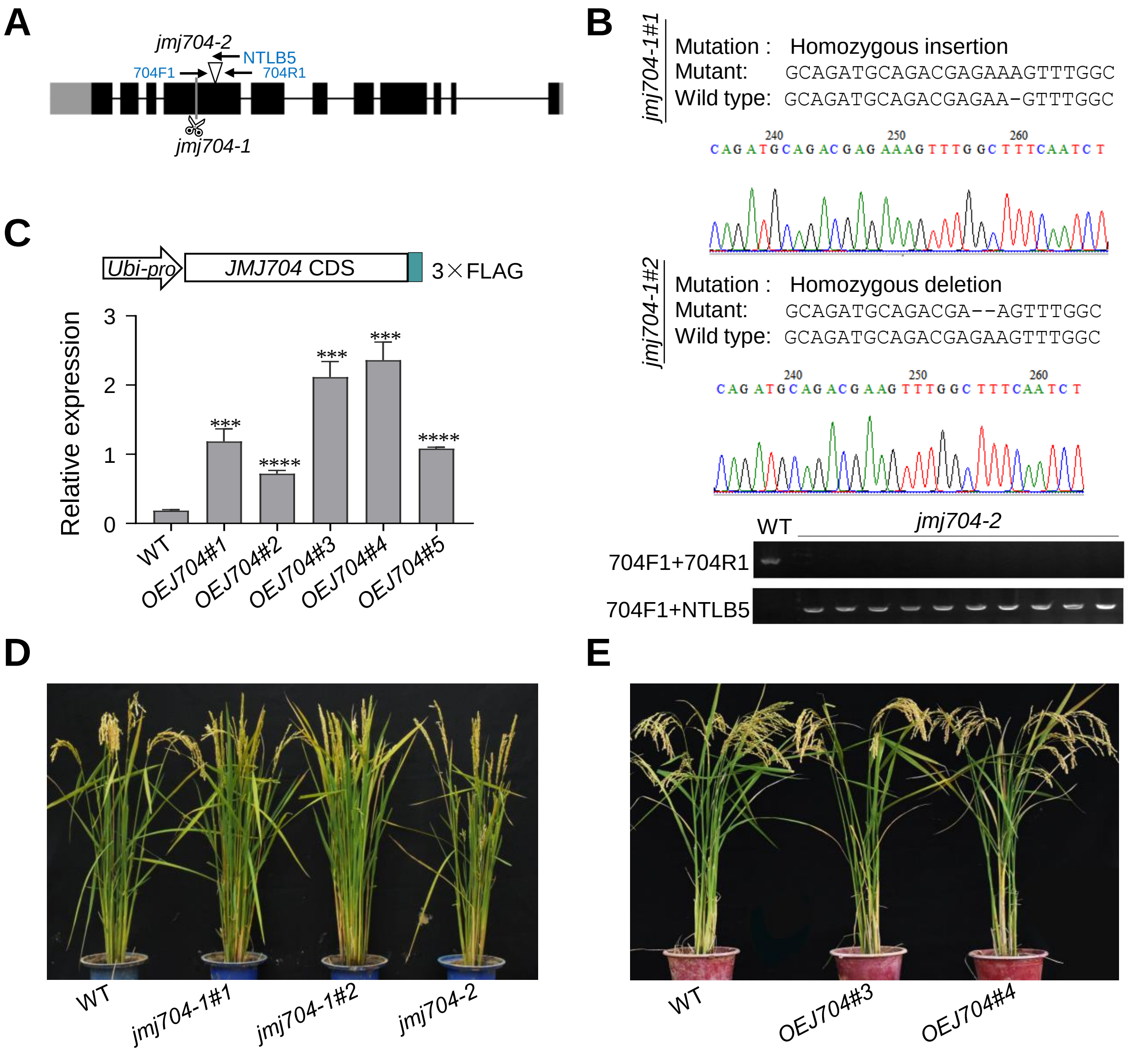

Figure S1. Characterization of *jmj704* mutant lines and *JMJ704* overexpression transgenic rice plants. (A) Gene structure of *JMJ704* and the CRISPR/Cas9 editing site and T-DNA insertion position in the *jmj704* mutants. **(B)** Genotypes of *jmj704* mutants (*jmj704-1*) generated by CRISPR/Cas9 system and the T-DNA mutant of *JMJ704* (*jmj704-2*). **(C)** Relative expression levels of *JMJ704* in the different overexpression lines. Data are means ± SD (n = 3). Significances were calculated using an unpaired two-tailed Student's t-test (***, $P < 0.001$; ****, $P < 0.0001$). **(D)** Phenotypes of *jmj704* mutants (*jmj704-1#1*, *jmj704-1#2*, *jmj704-2*) and **(E)** *JMJ704* overexpression plants (*OEJ704#3*, *OEJ704#4*) at grain-filling stage.

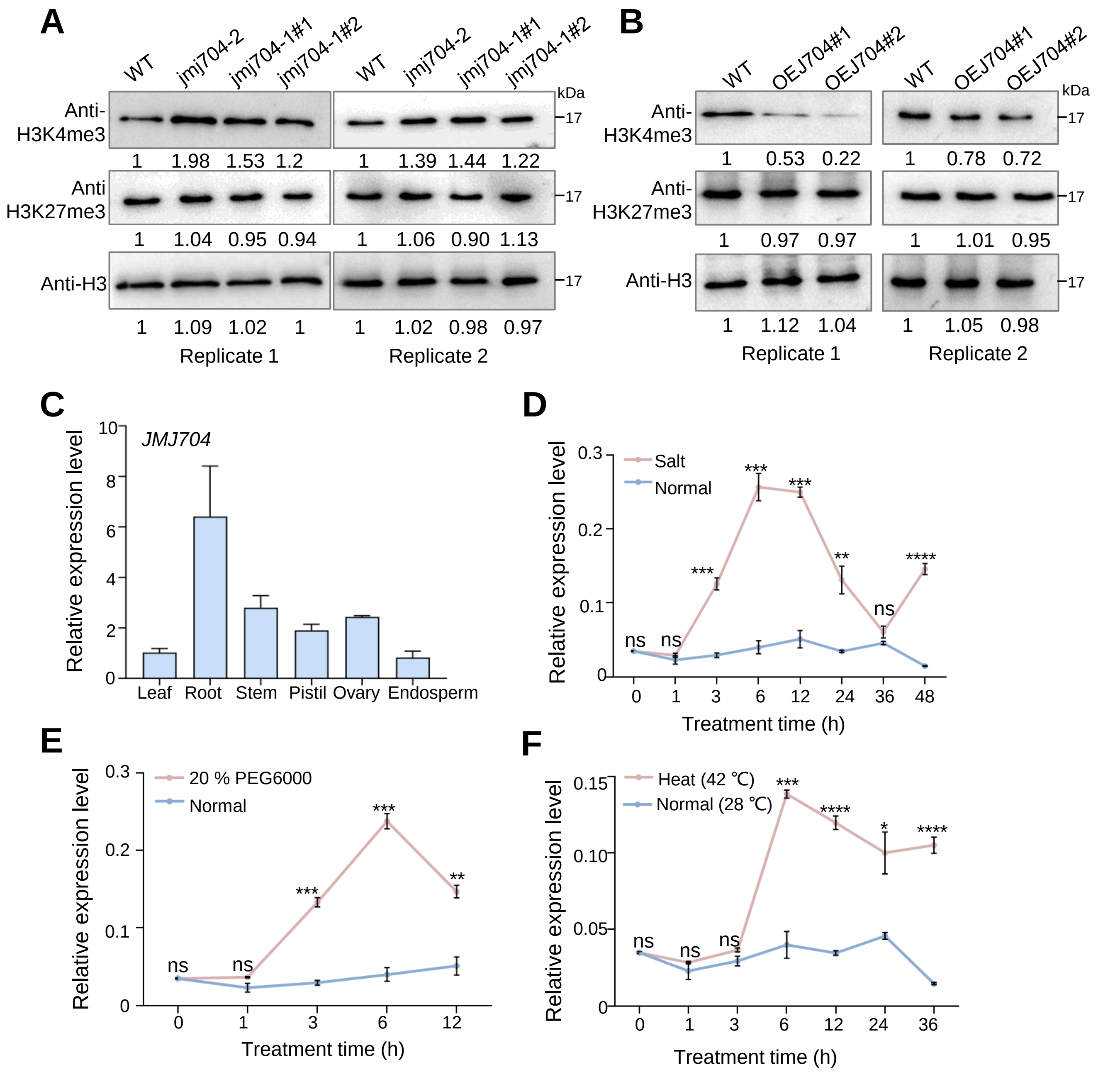

Figure S2. JMJ704 is an H3K4me3-specific histone demethylase involved in the response to multiple abiotic stresses in rice. (A-B) Immunoblotting detection of the enzyme activity of JMJ704 with H3K4me3 and H3K27me3 antibodies in *jmj704* mutant lines (**A**, *jmj704-1* and *jmj704-2*), *JMJ704* overexpression (*OEJ704*) plants **(B)**, and wild-type (WT). Two replicates are shown. The immunoblot signals were quantified using ImageJ software. **(C)** Relative expression levels of *JMJ704* in different rice tissues. *ACTIN* was used as the internal control. Data are means ± SD (n = 3). **(D-F)** Relative expression levels of *JMJ704* in wild-type rice seedlings under 150 mM NaCl **(D)**, 20% PEG6000 **(E)**, and 42 ℃ heat stress **(F)** treatments. *ACTIN* was used as an internal control. Data are means ± SD (n = 3). Significances were calculated using an unpaired two-tailed Student's t-test (ns, not significant; *, $P < 0.05$; **, $P < 0.01$; ***, $P < 0.001$; ****, $P < 0.0001$).

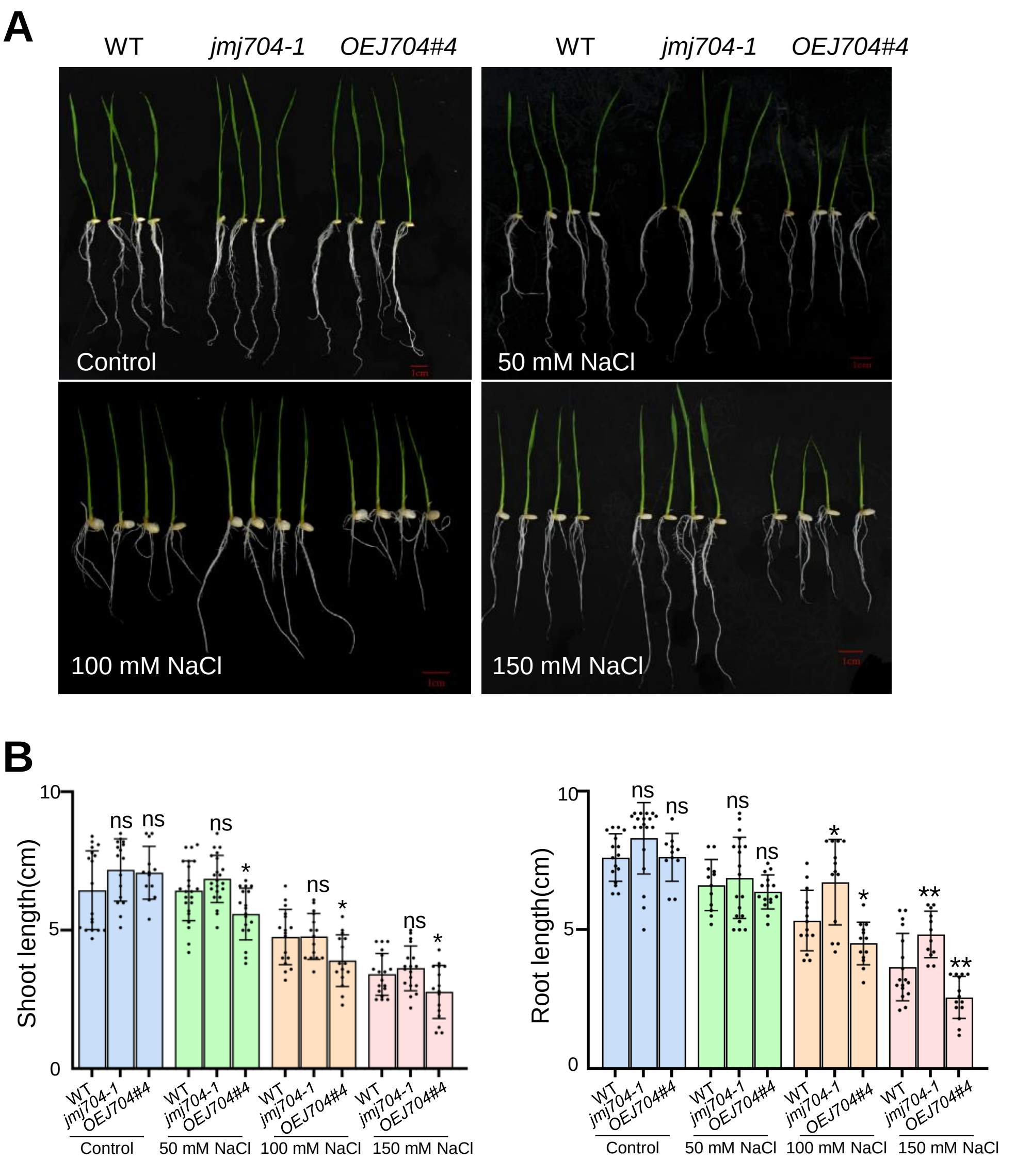

Figure S3. Effects of different salt concentrations on *JMJ704* overexpression and *jmj704* mutants of rice at seedling stages. (A) Phenotypes of *JMJ704* overexpression plants (*OEJ704#4*) and *jmj704* mutants (*jmj704-1#2*) growing in the ½ MS medium without NaCl (0 mM, control) or with different concentrations of NaCl (50 mM, 100 mM, 150 mM) for 7 days after germination for 2 days on normal medium. **(B)** Shoot and root length of the *OE-JMJ704* and *jmj704* mutants growing in the ½ MS medium with different salt concentrations. Data are means ± SD (*n* = 15). Significances were calculated using an unpaired two-tailed Student's *t*-test (ns, not significant; *, $P < 0.05$; **, $P < 0.01$).

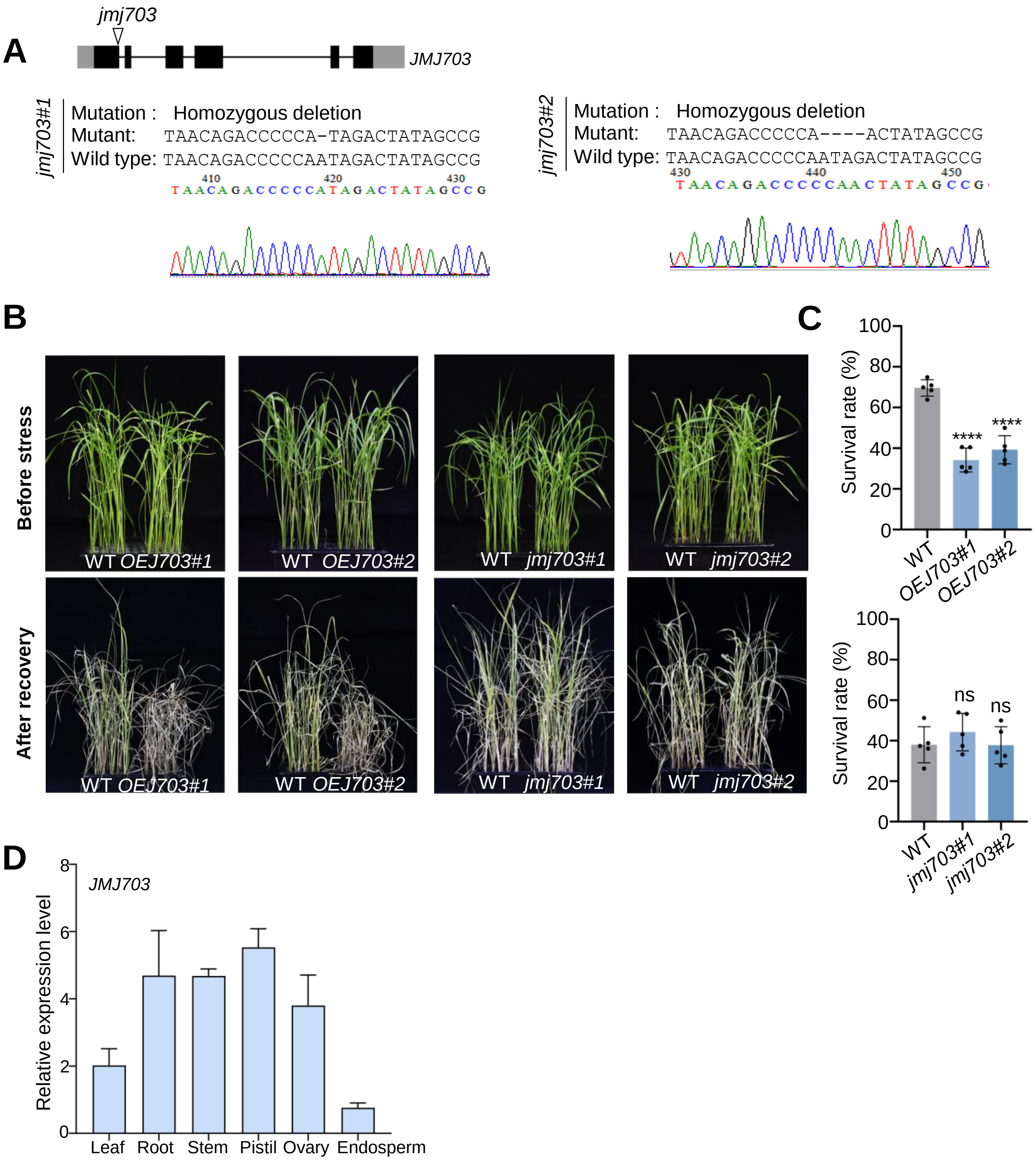

Figure S4. Effects of salt stress treatment on *JMJ703* overexpression plants and *jmj703* mutants in rice. (A) Genotypes of *jmj703* mutants (*jmj703#1* and *jmj703#2*) generated by CRISPR/Cas9 system. **(B)** Phenotypes of *OE-JMJ703* (*OEJ703#1* and *OEJ703#2*), *jmj703* mutants, and WT rice seedlings before and after salt stress treatment (150 mM NaCl treatment for 4-5 days, and recovery for 7 days). **(C)** Survival rates of the *OE-JMJ703* and *jmj703* mutants under salt stress. Data are means ± SD (n = 5). Significances were calculated using an unpaired two-tailed Student's t-test (ns, not significant; ****, $P < 0.0001$). **(D)** Relative expression levels of *JMJ703* in different rice tissues. *ACTIN* was used as the internal control. Data are means ± SD (n = 3).

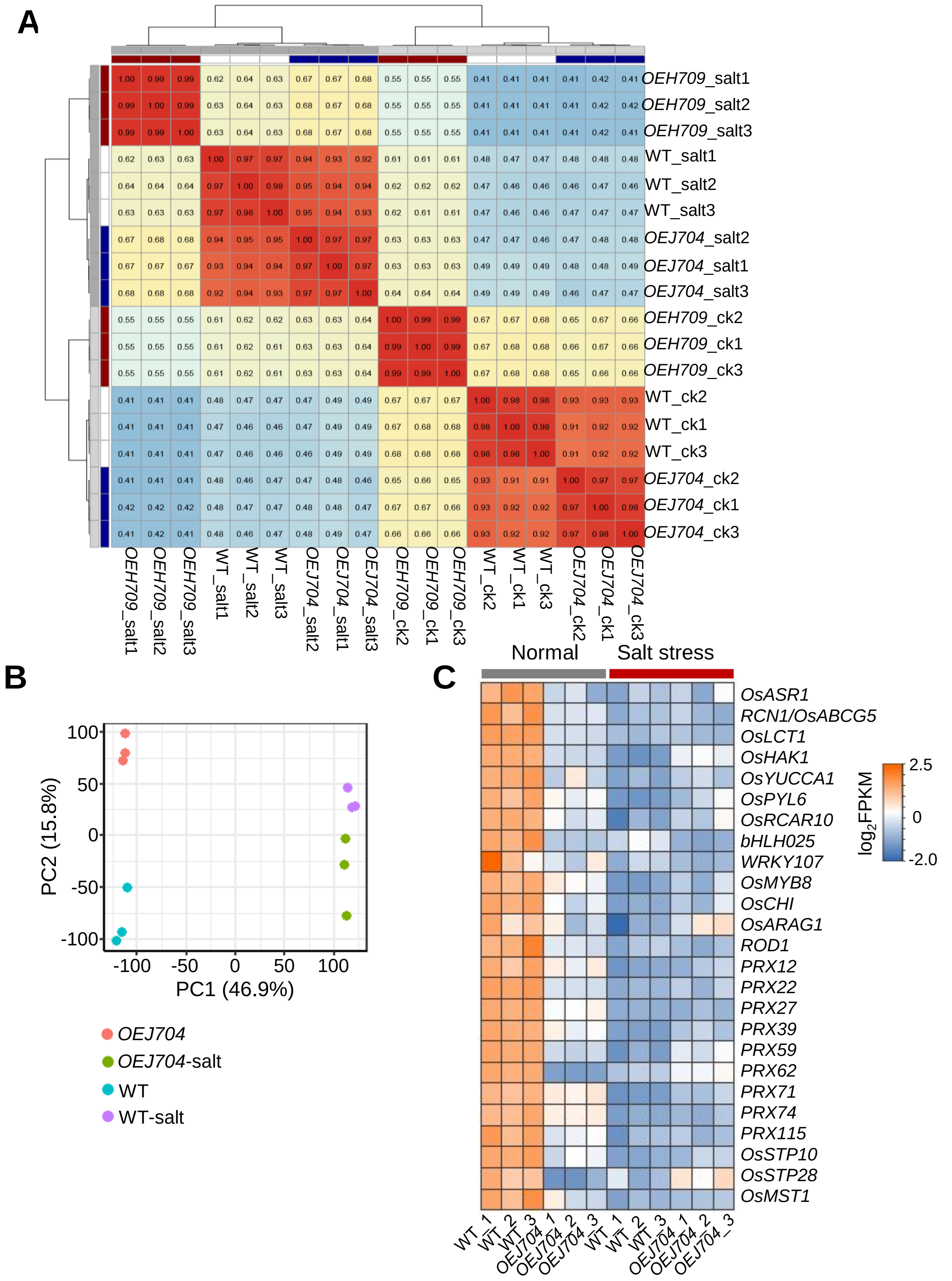

Figure S5. Analysis of RNA-seq data of *OE-JMJ704, OE-HDA709,* and WT rice seedlings under normal conditions and salt stress conditions. (A) Pearson correlation analysis of the RNA-seq data**. (B)** Principal component analysis of the transcriptomes of *OE-JMJ704* (*OEJ704*) and WT plants under normal conditions and salt stress conditions. **(C)** Heatmap showing that salt-responsive and oxidative stress-related genes are downregulated in *OE-JMJ704* plants.

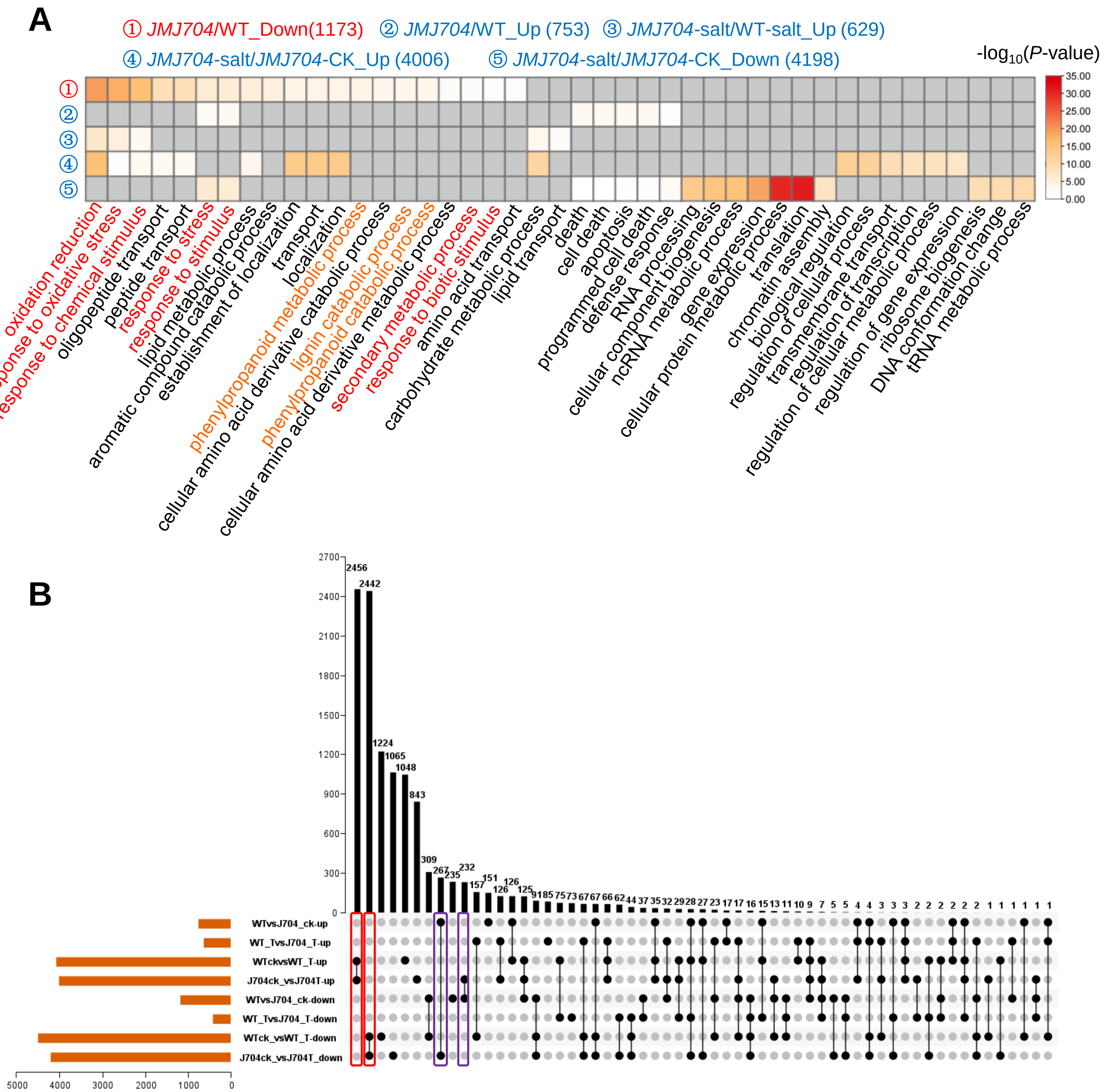

Figure S6. Analysis of differentially expressed genes (DEGs) identified between *OE-JMJ704* and WT plants under salt stress. (A) GO enrichment analysis of the DEGs in the five comparisons. Grey color indicates no GO terms enriched. **(B)** UpSet plots showing the overlapping genes among the upregulated and downregulated genes in *OE-JMJ704* versus WT under normal conditions and salt stress conditions.

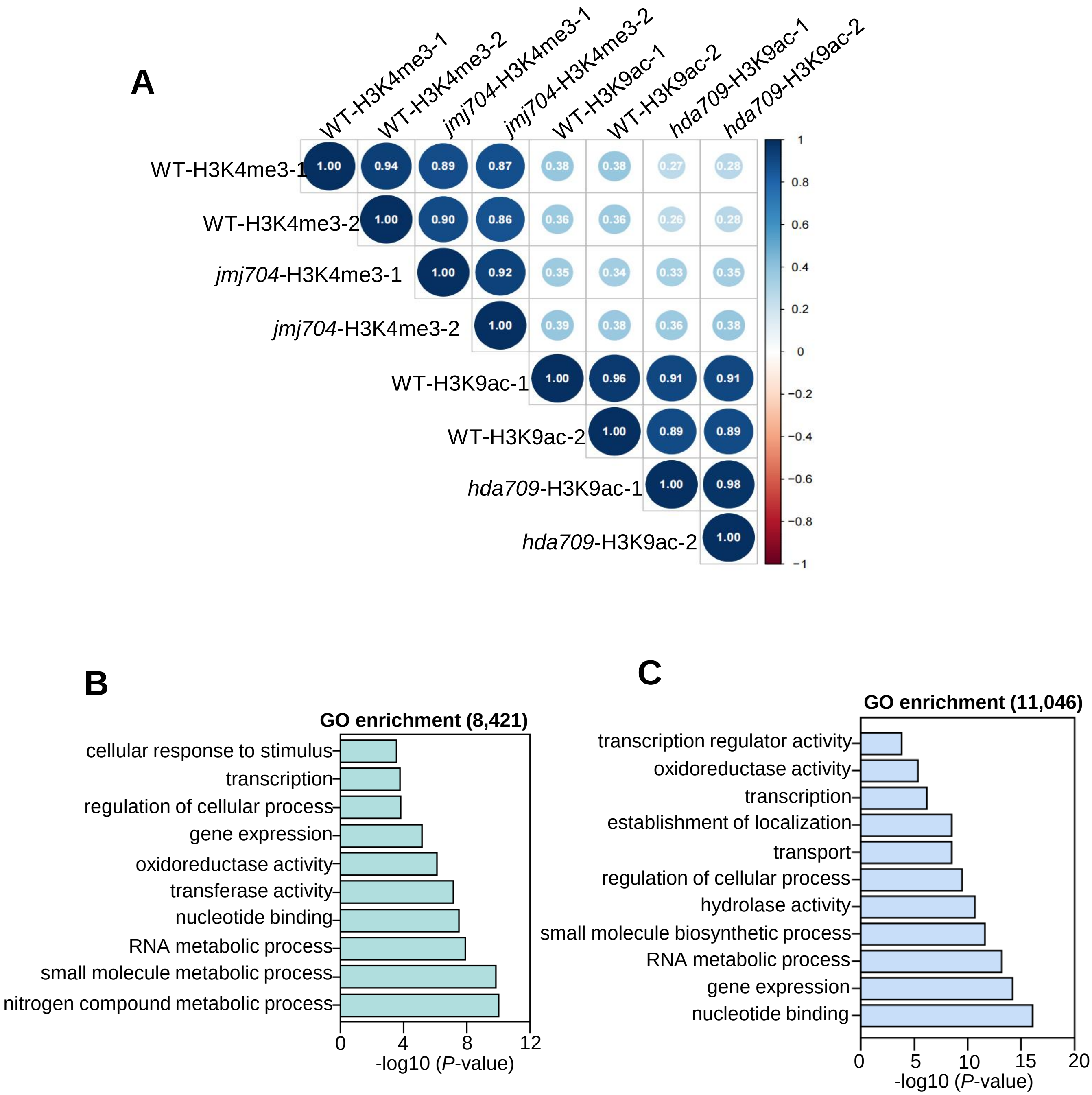

Figure S7. Analysis of the ChIP-seq data of *jmj704 mutant*, *hda709 mutant,* and WT rice seedlings. (A) Correlation analysis of H3K4me3 (in WT and *jmj704*) and H3K9ac (in WT and *hda709*) ChIP-seq datasets. **(B)** GO enrichment analysis of the H3K4me3 hyper-methylated genes in the *jmj704* mutant. **(C)** GO enrichment analysis of the H3K9ac hyper-acetylated genes in the *hda709* mutant.

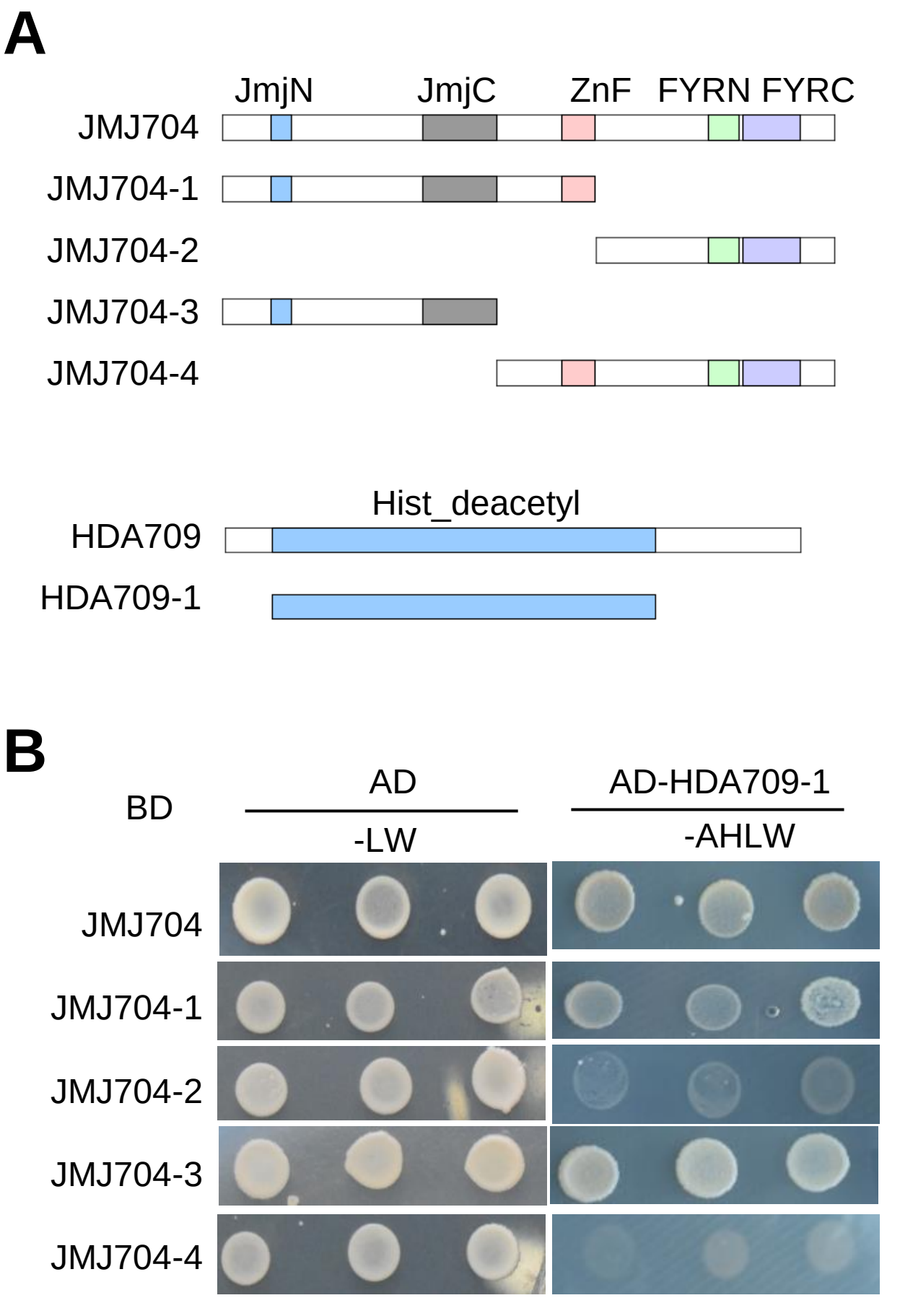

Figure S8. Y2H assay of interaction between the truncated regions of JMJ704 and HDA709. (A) Schematic structures of full length and truncated domains of JMJ704 and HDA709. **(B)** Tests of JMJ704 fragments for interaction with HDA709. Full length JMJ704 (amino acids [aa] 1-971), JMJ704-1 (aa 1-591), JMJ704-2 (aa 592-971), JMJ704-3 (aa 1-434), and JMJ704-4 (aa 435-971) were fused to the GAL4-BD (binding domain) as indicated. Full length HDA709 (aa 1-456) and HDA709-1 (aa 38-339) were fused to the GAL4-AD (activation domain) as indicated. Yeast cells were spotted onto a stringent selection medium lacking Trp, Leu, His, and Ade (-WLHA) or a nonselective medium lacking Trp and Leu (-WL) as a control.

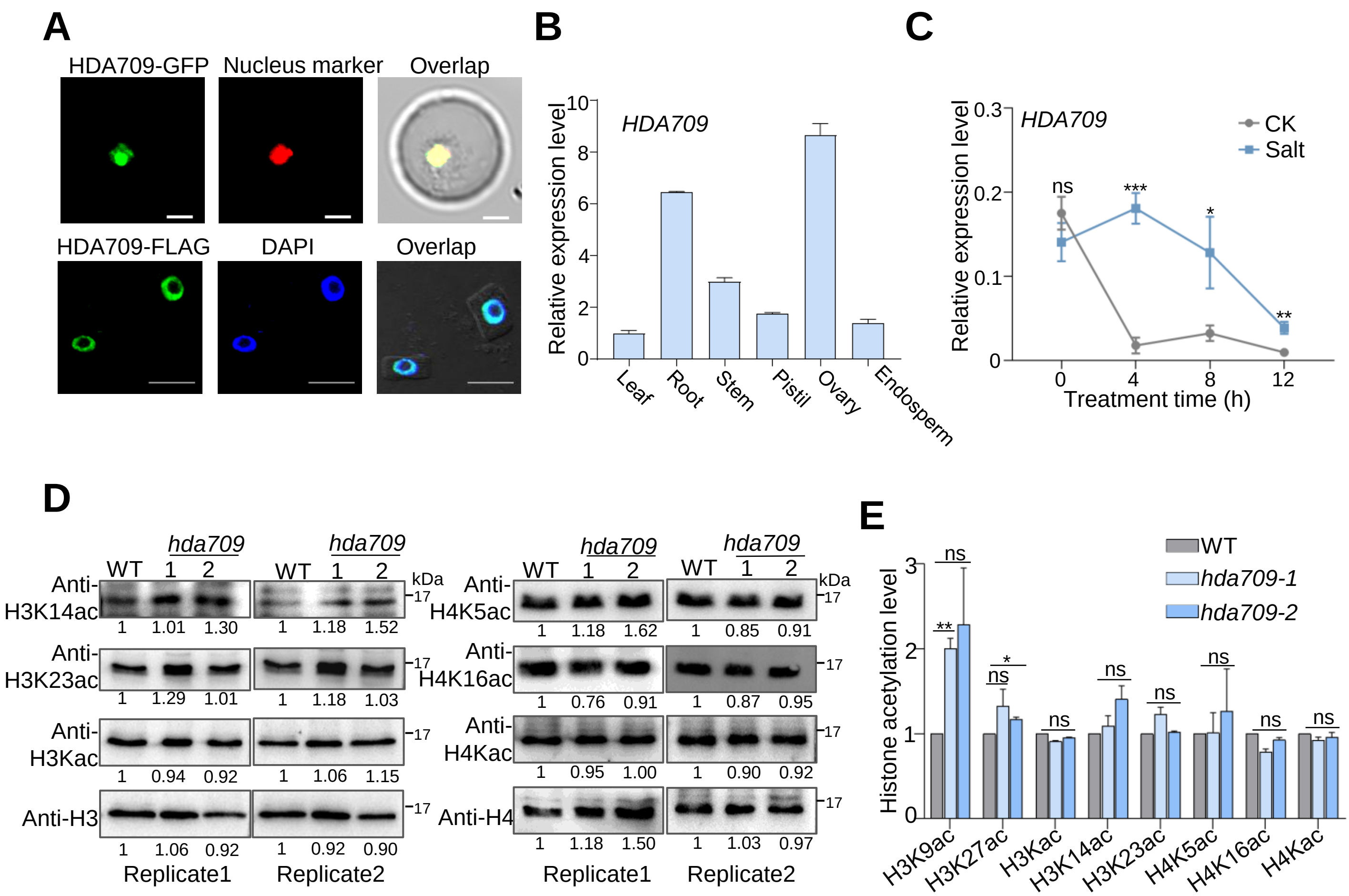

Figure S9. Analysis of subcellular localization and molecular function of HDA709. (A) Subcellular localization of HDA709 in rice. Upper panel indicates the subcellular localization of HDA709 observed in rice protoplasts. Lower panel shows the nuclear localization detected by immunostaining with an anti-FLAG antibody in rice root cells. Scale bars = 5 μm. **(B)** Relative expression levels of *HDA709* in different rice tissues. *ACTIN* was used as the internal control. Data are means ± SD (*n* = 3). **(C)** Relative expression levels of *HDA709* in wild-type rice seedlings treated with 150 mM NaCl for the indicated time points. *ACTIN* was used as the internal control. Data are means ± SD (*n* = 3). Significant differences compared with corresponding controls were determined using an unpaired two-tailed Student's *t*-test. **(D)** Immunoblotting analysis of histone acetylation levels in WT and *hda709* (*hda709-1* and *hda709-2*) mutant lines. **(E)** Quantitative analyses of the immunoblot signal intensities from (D) and Figure 4A. Histone H3 or H4 was used as the loading control. The immunoblot signals were quantified using ImageJ software. Data are means ± SD from two independent biological replicates. Significances compared with WT were calculated using an unpaired two-tailed Student's *t*-test (ns, not significant; *, $P < 0.05$; **, $P < 0.01$; ***, $P < 0.001$).

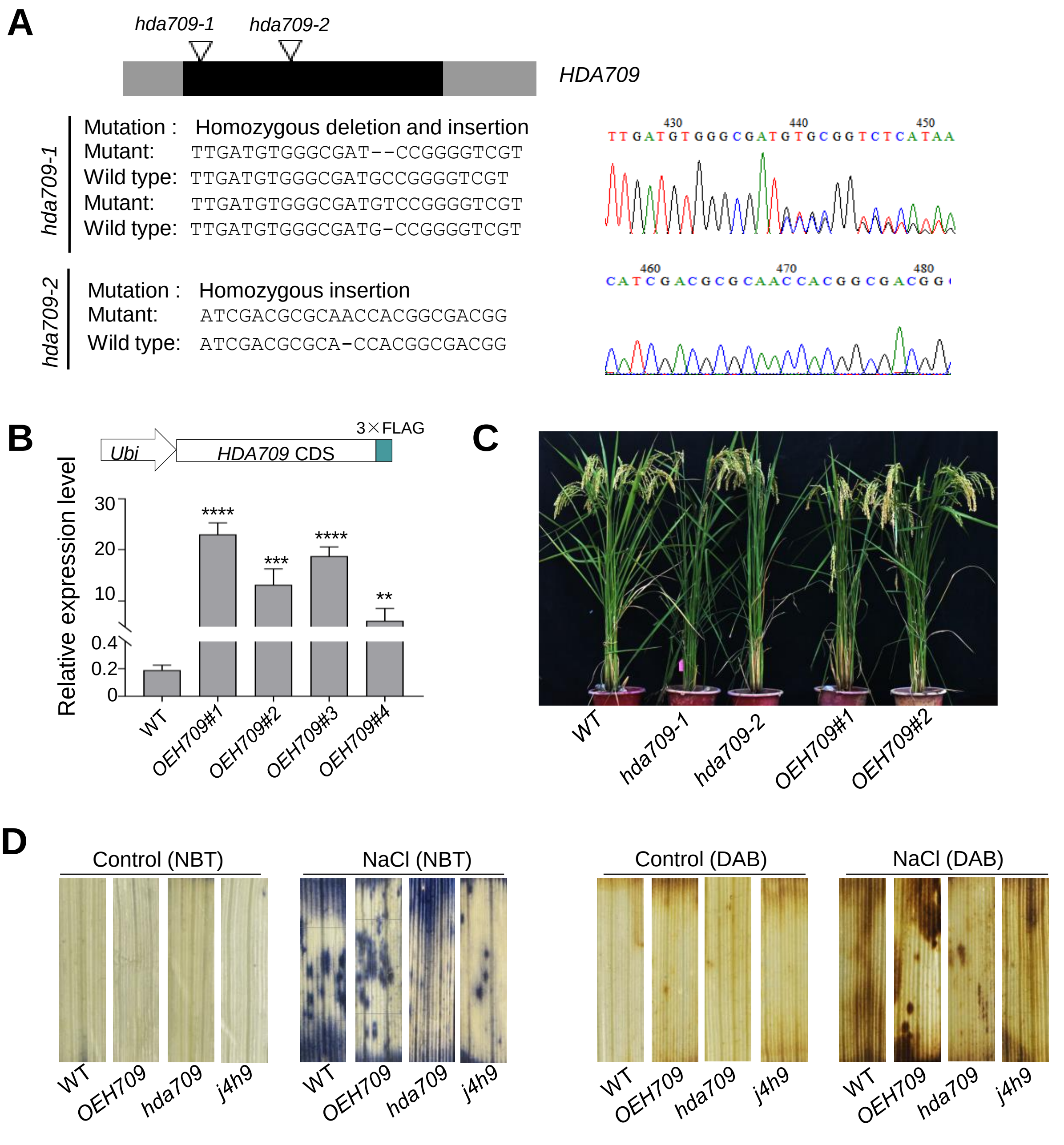

Figure S10. Characterization of *HDA709* overexpression plants and *hda709* mutant lines of rice. (A) Genotypes of *hda709* mutants (*hda709-1, hda709-2*) generated by the CRISPR/Cas9 system. **(B)** Relative expression levels of *HDA709* in different *OE-HDA709* transgenic lines of rice. Data are means ± SD (n = 3). Significances were calculated using an unpaired two-tailed Student's t-test (**, P < 0.01; ***, P < 0.001; ****, P < 0.0001). **(C)** Phenotypes of *hda709* mutants (*hda709-1, hda709-2*) and *HDA709* overexpression lines (*OEH709#1, OEH709#2*) of rice at grain-filling stages. **(D)** DAB and NBT staining showing the ROS contents in leaves of WT, *OE-HDA709*, *hda709*, and *jmj704hda709* (*j4h9*) under normal conditions and salt stress (150 mM NaCl, 24 h).

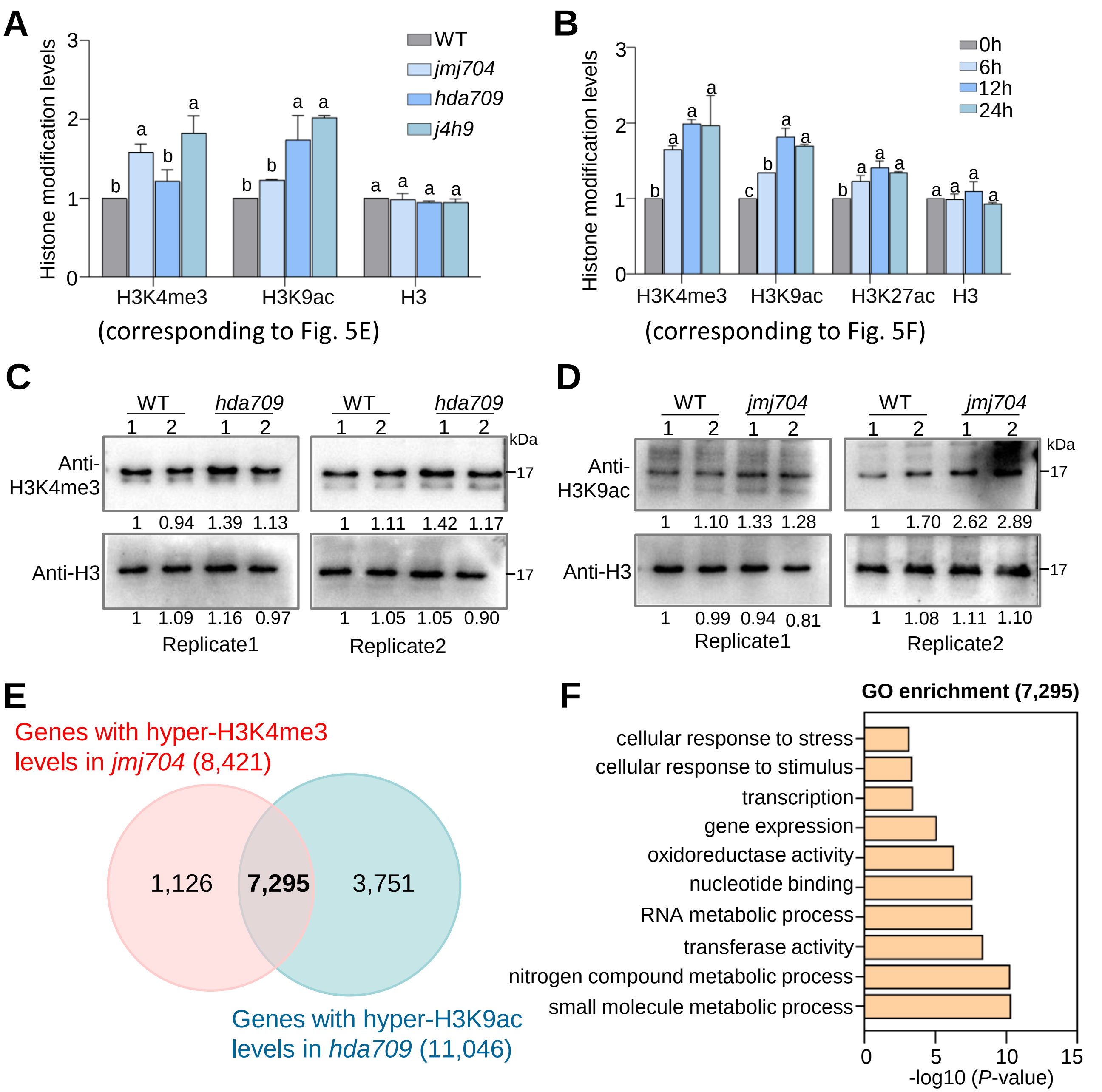

Figure S11. Histone modifications crosstalk between H3K4me3 and H3K9ac mediated by JMJ704 and HDA709, respectively. (A) Quantification of H3K4me3 and H3K9ac levels in WT, *jmj704*, *hda709*, and *jmj704hda709* (*j4h9*) double mutants, corresponding to Figure 5E. **(B)** Quantification of the dynamic changes of histone modifications during time-course salt stress, corresponding to Figure 5F. The immunoblot signals were quantified using ImageJ software. Data are means ± SD from two independent biological replicates. Significant differences ($P < 0.05$) are indicated by different letters based on one-way ANOVA followed by Tukey's multiple comparison test. **(C)** Immunoblot assay of the H3K4me3 levels in the *hda709* mutants. **(D)** Immunoblot assay of the H3K9ac levels in the *jmj704* mutants. Two replicates are shown. The immunoblot signals were quantified using ImageJ. **(E)** Venn diagram showing the overlapping genes with hyper-H3K9ac and hyper-H3K4me3 levels in the *hda709* and *jmj704* mutants. **(F)** GO enrichment analysis of the overlapping genes from (E).

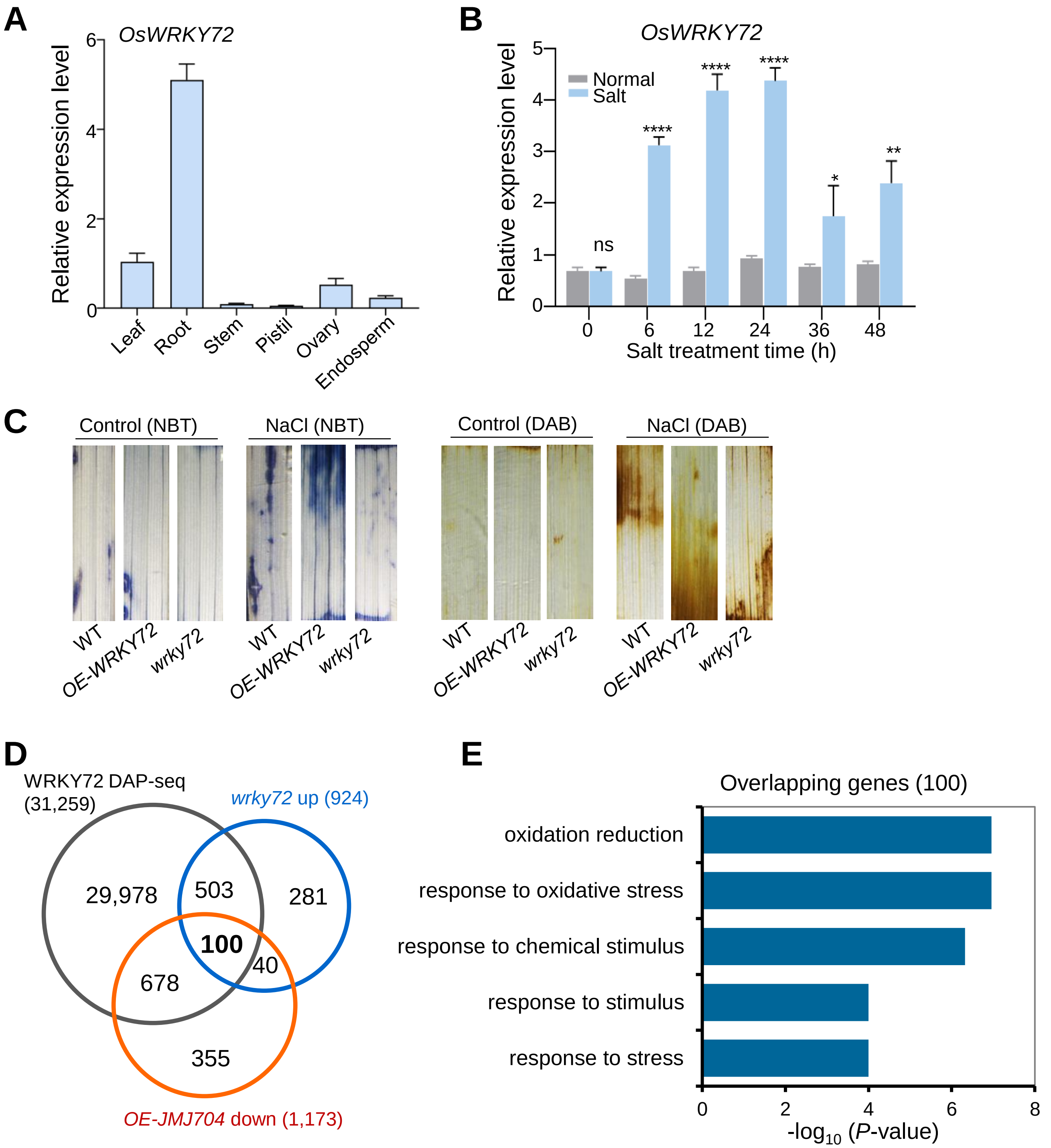

Figure S12. Identification of the common target genes of OsWRKY72 and JMJ704 in rice. (A) Relative expression levels of *OsWRKY72* in various rice tissues. *Actin* was used as the internal control. Data are means ± SD (n = 3). **(B)** Expression levels of *OsWRKY72* in wild-type rice seedlings during different salt treatment times. Data are means ± SD (n = 3). Significances were calculated using an unpaired two-tailed Student's t-test (ns, not significant; *, $P < 0.05$; **, $P < 0.01$; ****, $P < 0.0001$). **(C)** NBT and DAB staining assays showing ROS accumulation in leaves of WT, *OE-WRKY72*, and *wrky72* under normal conditions and salt stress (150 mM NaCl, 24 h). **(D)** Venn diagram showing the overlapping genes among OsWRKY72 binding genes and the downregulated genes in *OE-JMJ704* and upregulated genes in *wrky72*. **(E)** GO enrichment analysis of the common target genes of OsWRKY72 and JMJ704.

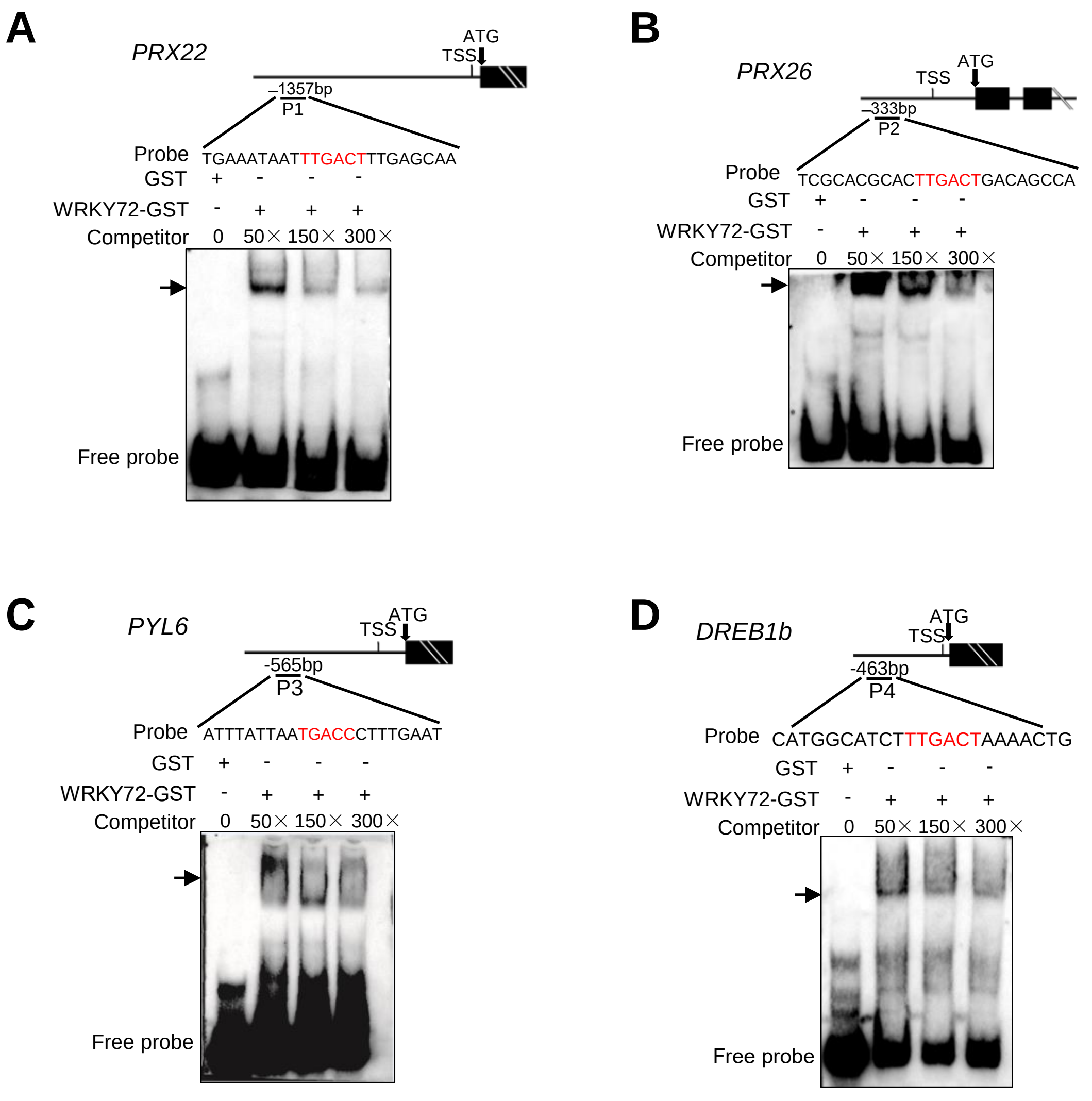

Figure S13. EMSA experiments showing OsWRKY72 binding to the promoters of *PRX22, PRX26, PYL6,* and *DREB1b*. Electrophoretic mobility shift assay (EMSA) of OsWRKY72 binding to the (T)TGAC(C/T) motif (OsWRKY72-binding site) in the promoters of *PRX22* **(A)**, *PRX26* **(B)**, *PYL6* **(C)**, and *DREB1b* **(D)**. *E. coli*-produced OsWRKY72 protein was incubated with 5'-biotin-labeled promoter fragments of its target genes in the absence or presence of 50-, 150-, or 300-fold excess of the corresponding cold probes and analyzed by electrophoresis. The shifted bands are indicated by the arrow.

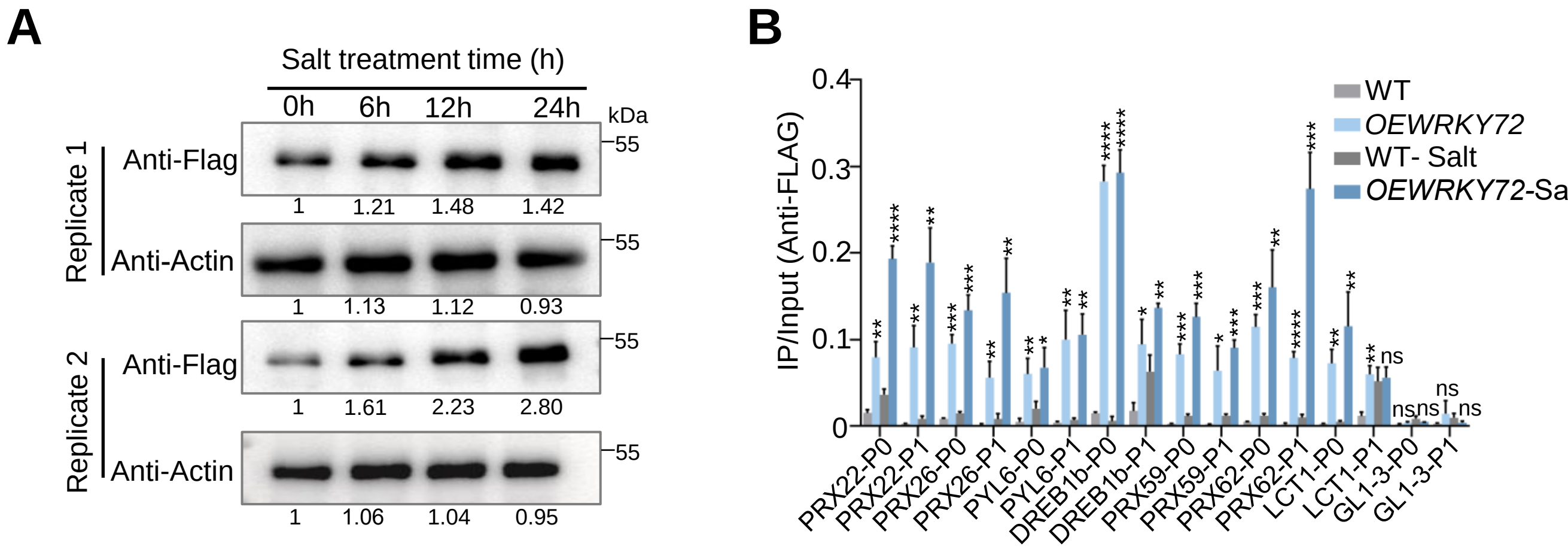

Figure S14. Salt stress promotes OsWRKY72 protein accumulation and enhances its transcriptional activity and DNA-binding capacity. (A) Immunoblotting analysis showing the OsWRKY72 protein levels in *OE-WRKY72* plants during salt stress treatment. *ACTIN* was used as the loading control. **(B)** ChIP-qPCR analysis of OsWRKY72 binding to the promoters of *PRX22, PRX26, PYL6, DREB1b, PRX59, PRX62,* and *LCT1* in WT and *OE-WRKY72* plants under normal conditions and salt stress conditions, using an anti-FLAG antibody for immunoprecipitation. Data are means ± SD (n = 3). Significances were calculated using an unpaired two-tailed Student's t-test (ns, not significant; *, $P < 0.05$; **, $P < 0.01$; ***, $P < 0.001$; ****, $P < 0.0001$).

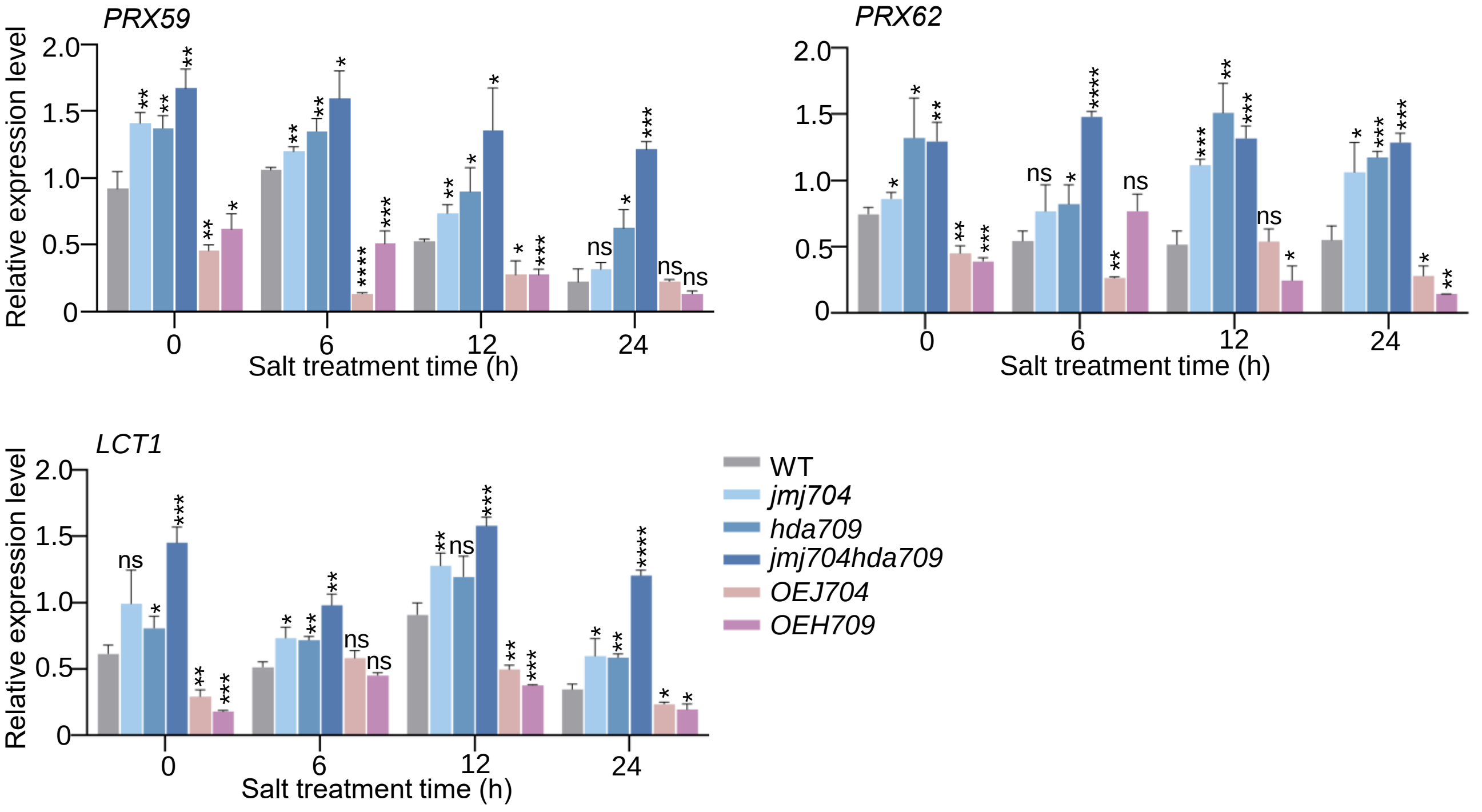

Figure S15. Relative expression levels of *PRX59, PRX62* and *LCT1* in *OE-JMJ704* (*OEJ704*), *jmj704* mutant, *OE-HDA709* (*OEH709*), *hda709* mutant, *jmj704hda709* double mutants and WT plants under salt stress treatment for different times (150 mM NaCl treatment for 0 [control], 6, 12, 24 hours). Data are means ± SD (n = 3). Significances were calculated using an unpaired two-tailed Student's *t*-test (ns, not significant; *, $P < 0.05$; **, $P < 0.01$; ***, $P < 0.001$; ****, $P < 0.0001$).

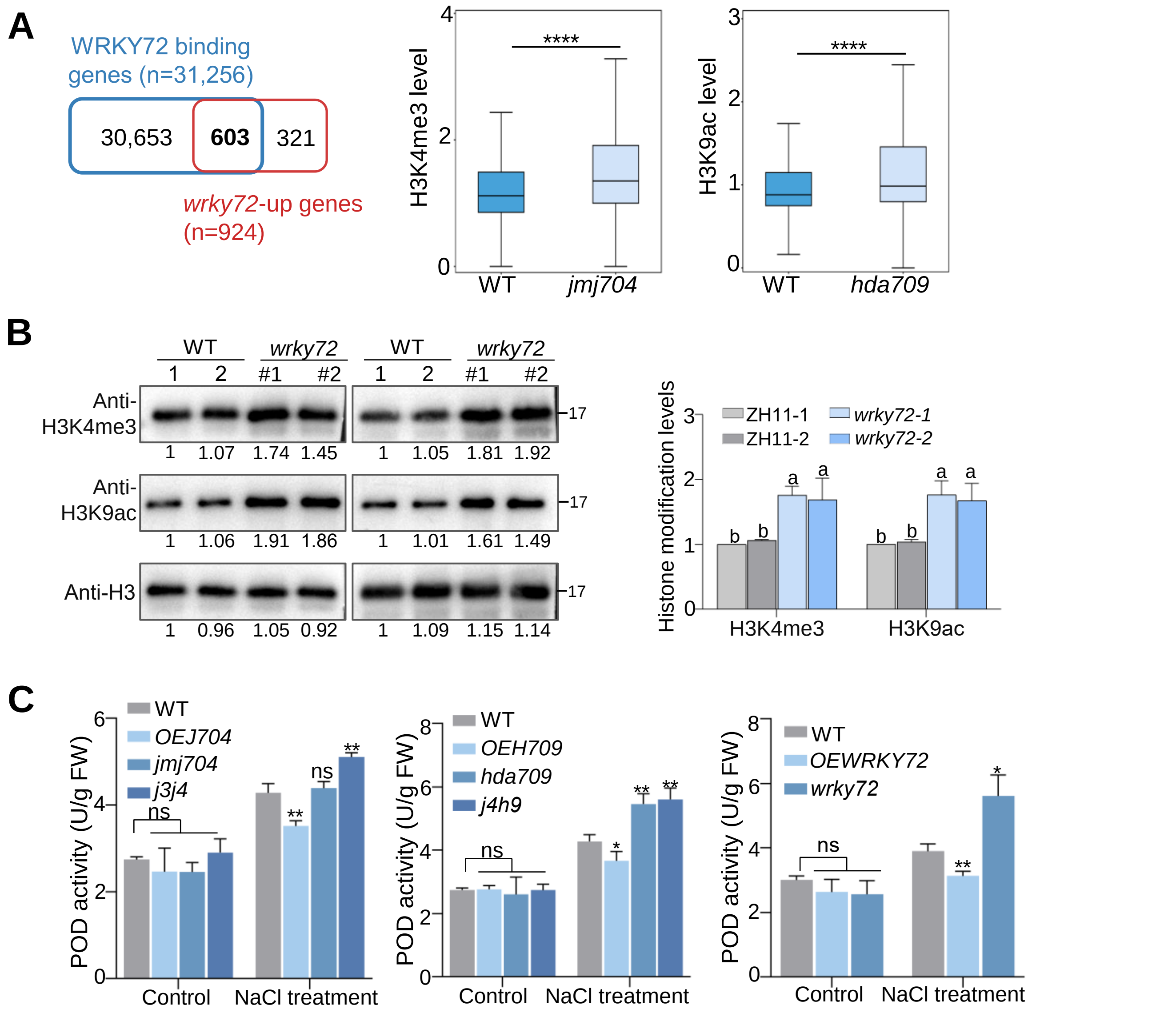

Figure S16. Genome-wide analysis of OsWRKY72 targets and associated histone modifications. (A) Venn diagram showing the overlapping genes between OsWRKY72 binding genes and the upregulated genes in *wrky72* (left). Boxplot showing the H3K4me3 and H3K9ac levels of these 603 target genes in the *jmj704, hda709,* and WT plants (right). **(B)** Analysis of histone H3K4me3 and H3K9ac levels in WT and two *wrky72* mutant lines (*wrky72#1* and *wrky72#2*) by immunoblotting using anti-H3K4me3 and anti-H3K9ac antibodies. Total histone extracts from 14-day-old seedlings were used for the analysis. Data are means ± SD from two independent biological replicates. One-way ANOVA with Tukey's multiple comparison test was performed for statistical analysis. Significant differences ($P < 0.05$) are indicated by different letters. **(C)** POD activity assays in *OE-JMJ704, jmj704, jmj703jmj704 (j3j4), OE-HDA709, hda709, jmj704hda709 (j4h9), OE-WRKY72*, *wrky72,* and WT plants under normal conditions and salt stress (150 mM NaCl, 24 h). Data are means ± SD (n = 3). Significances were calculated using an unpaired two-tailed Student's t-test (ns, not significant; *, $P < 0.05$; **, $P < 0.01$; ****, $P < 0.0001$).
