# Supplementary material for: Histone Modification Complex JMJ704‐HDA709 Negatively Regulates Salinity Tolerance in Rice
Source: Adv Sci (Weinh). 2026 Jun 1:e75873. Online ahead of print. doi: 10.1002/advs.75873 (PMC13335786; doi:10.1002/advs.75873)

Figure 3 source data

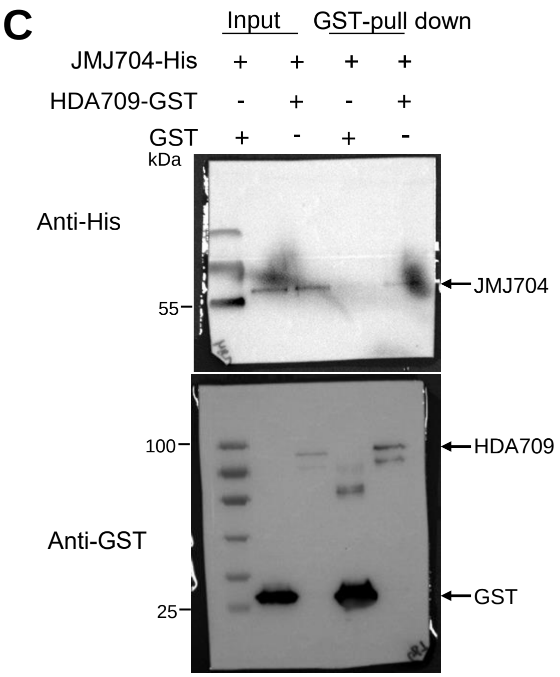

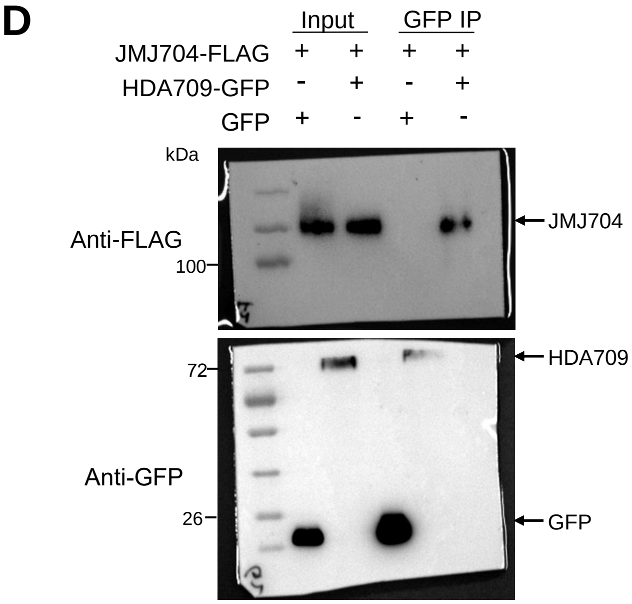

Figure 4 source data

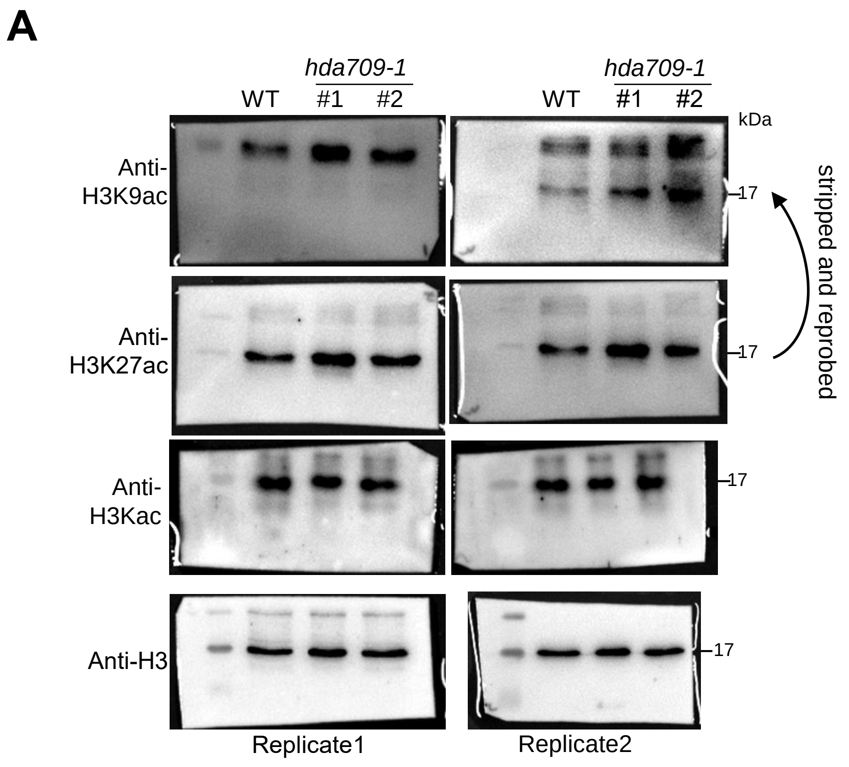

Figure 5 source data

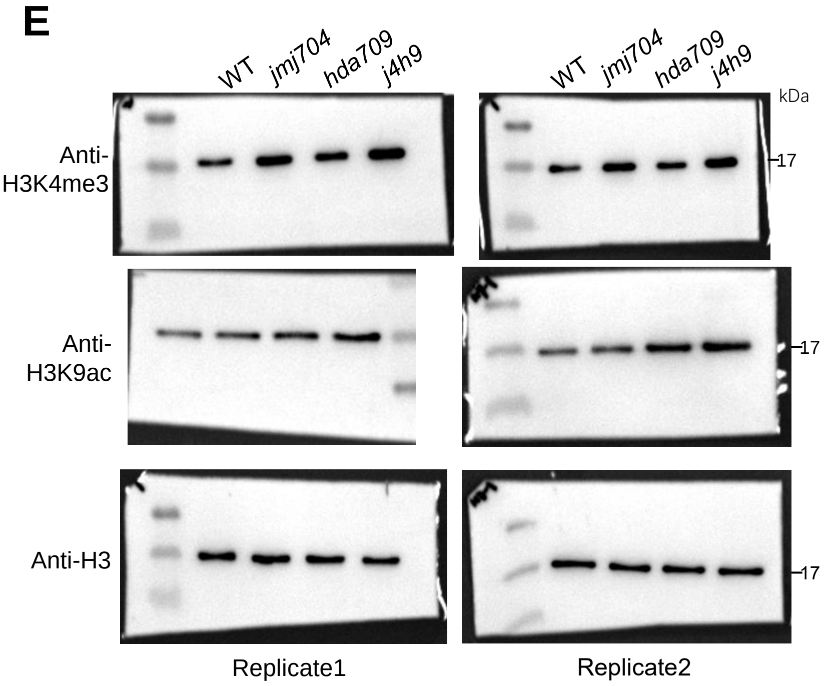

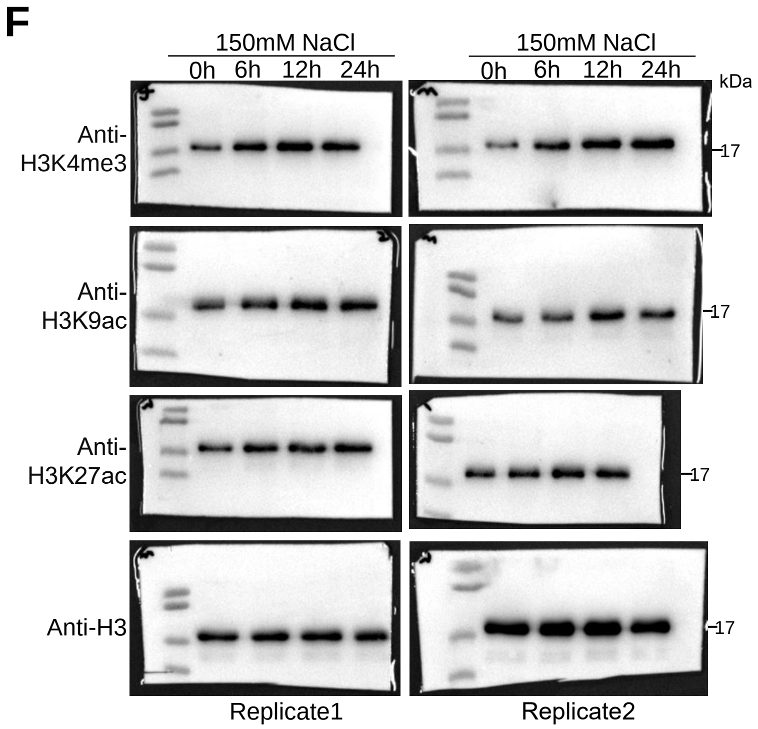

Figure 6 source data

B

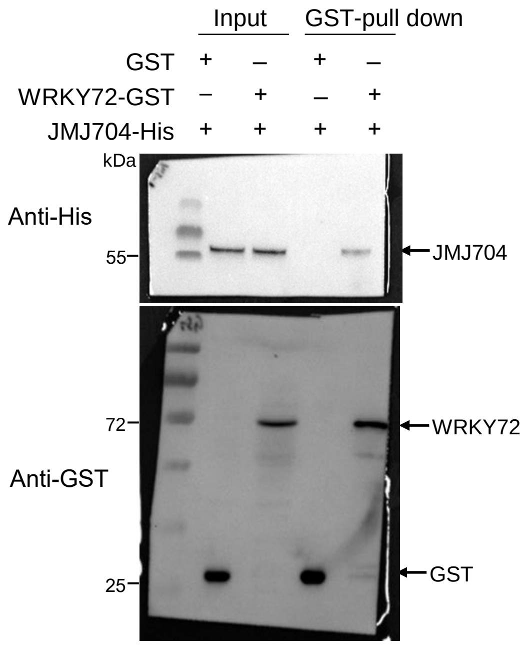

D

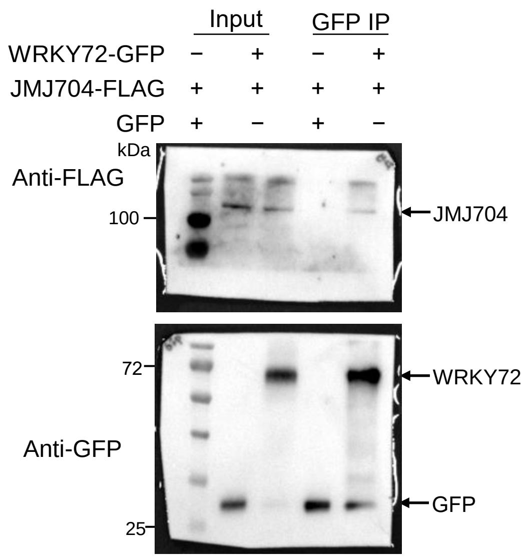

E

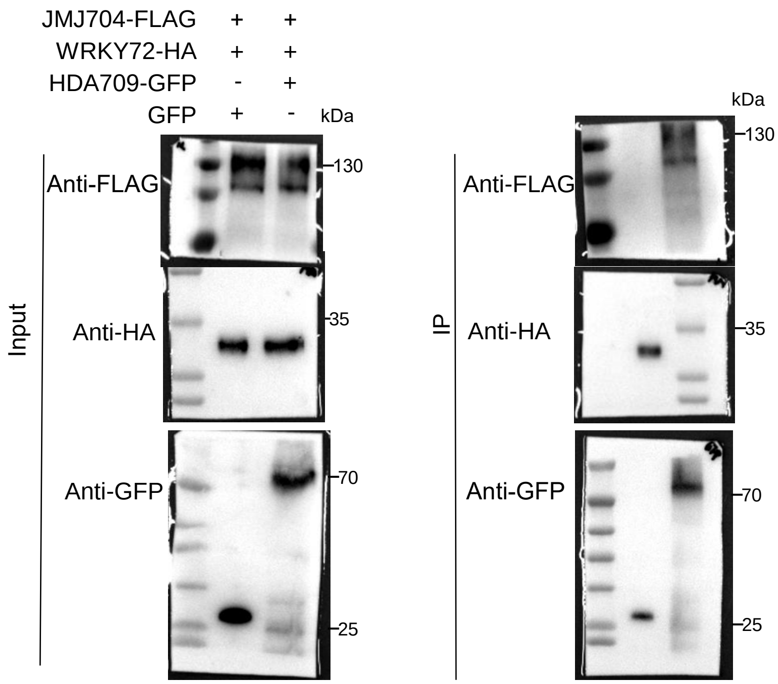

Figure 7 source data

A

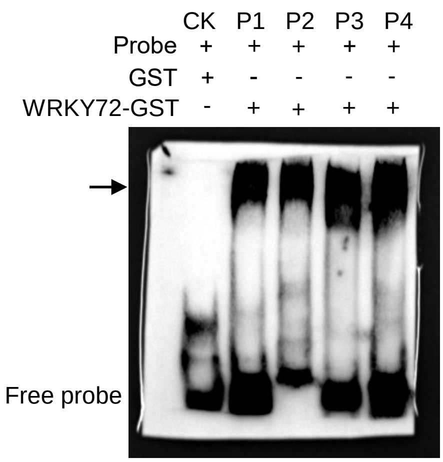

Figure S2 source data

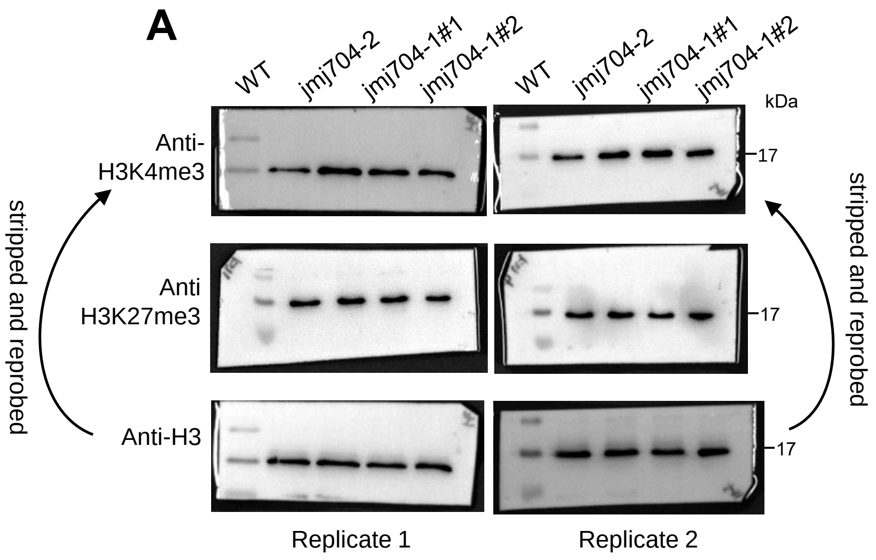

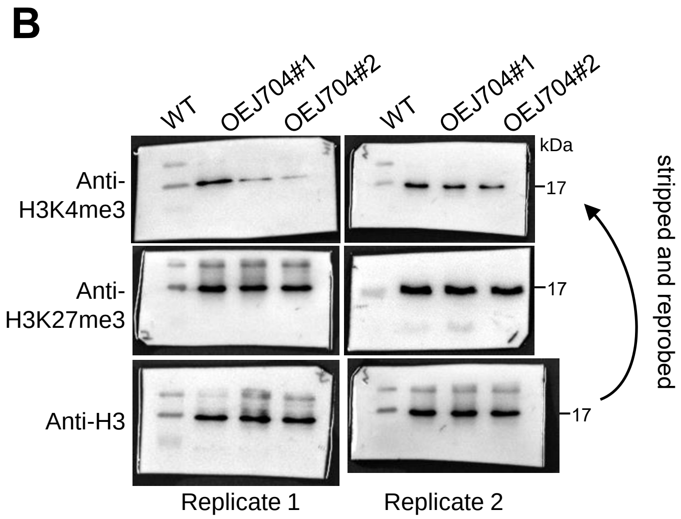

Figure S9 source data

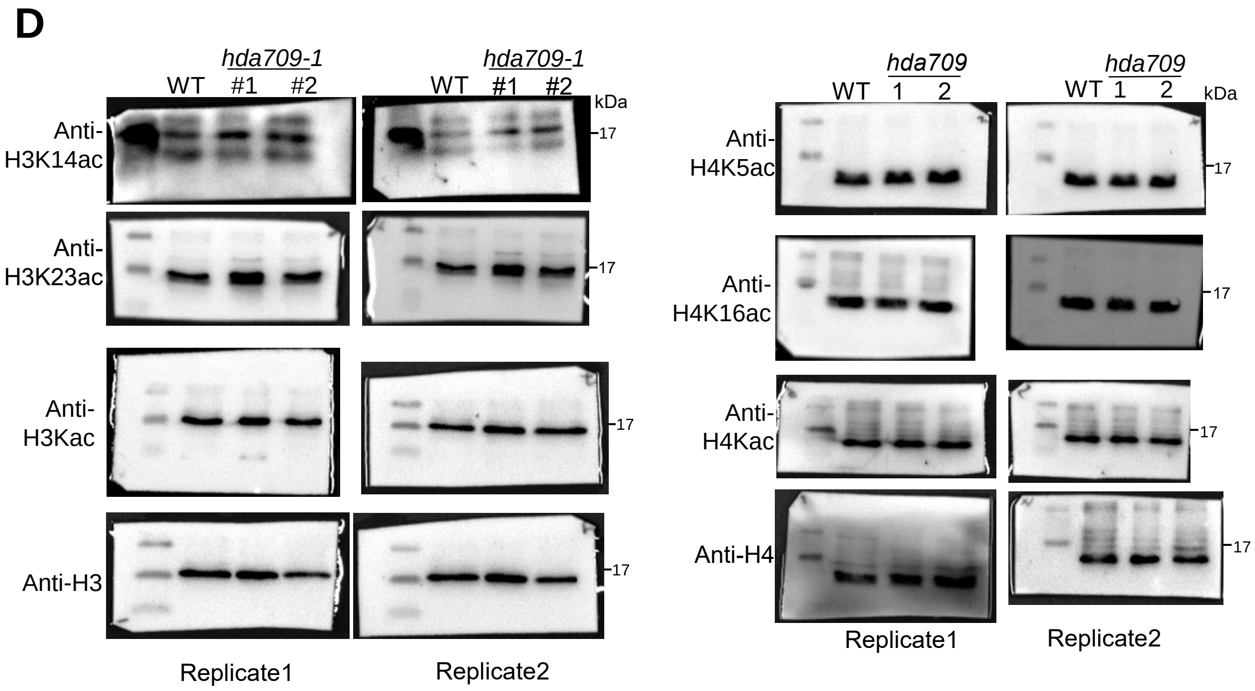

Figure S11 source data

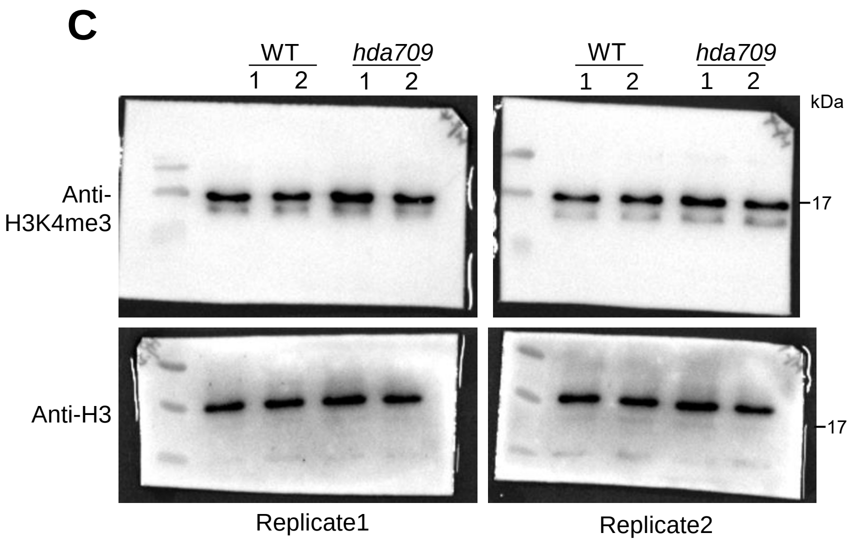

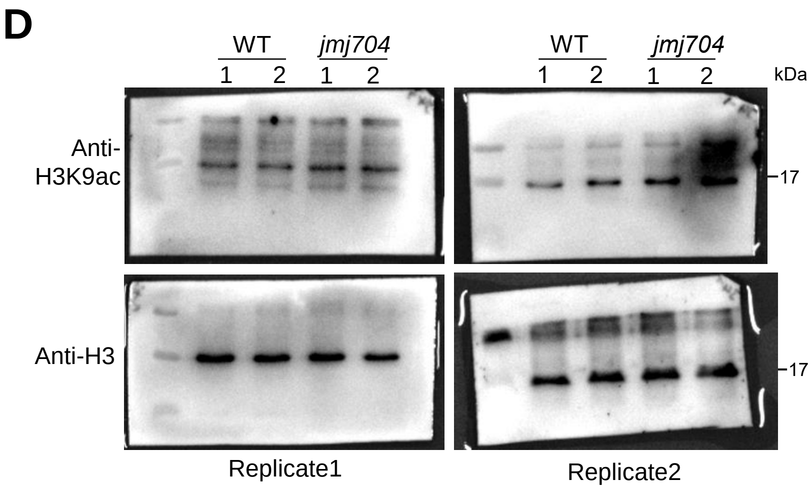

Figure S13 source data

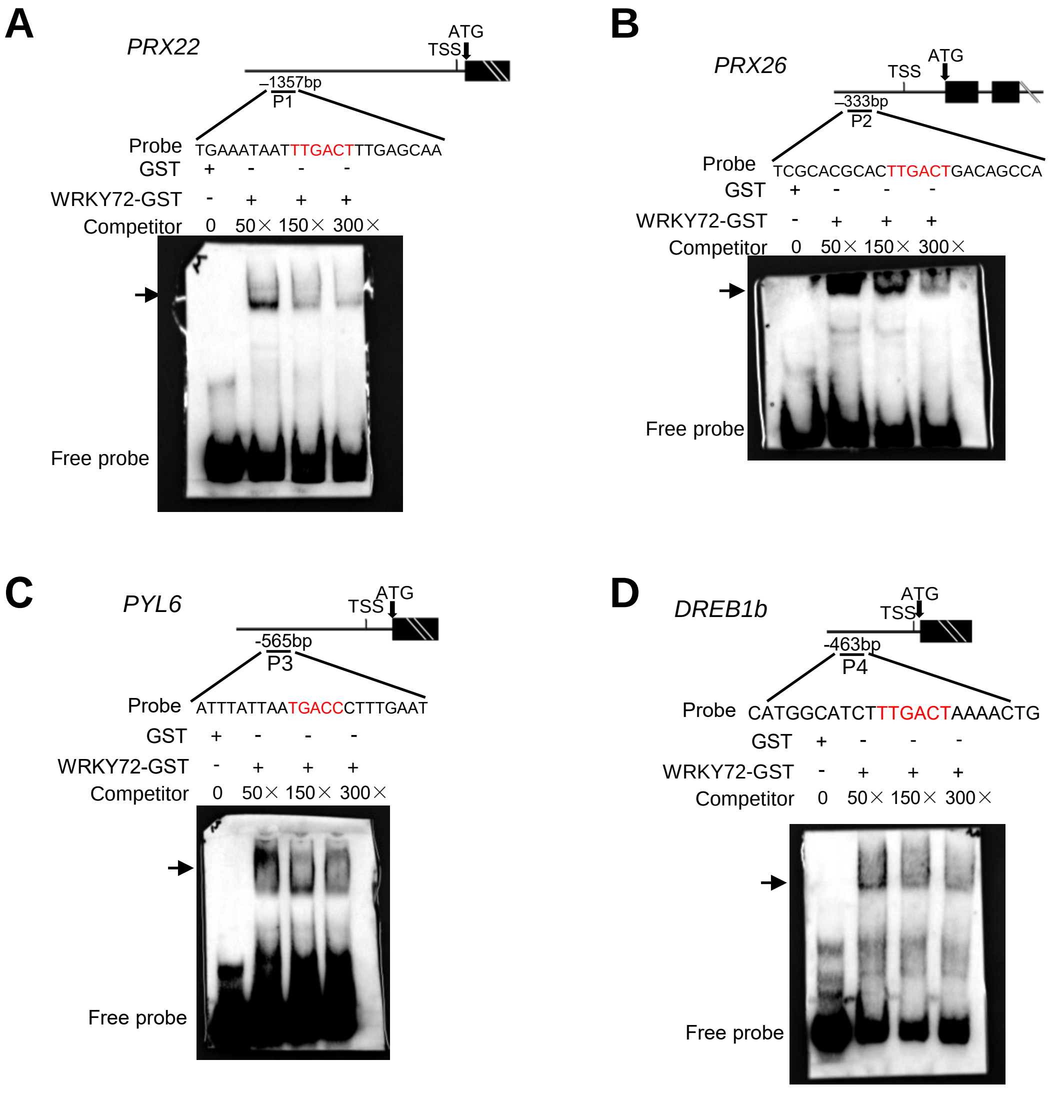

Figure S14 source data

A

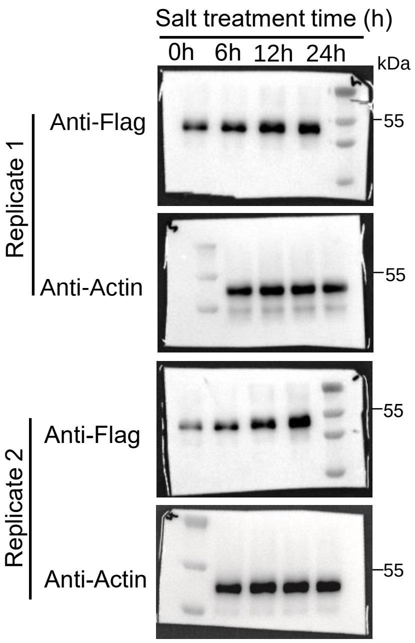

Figure S16 source data

B

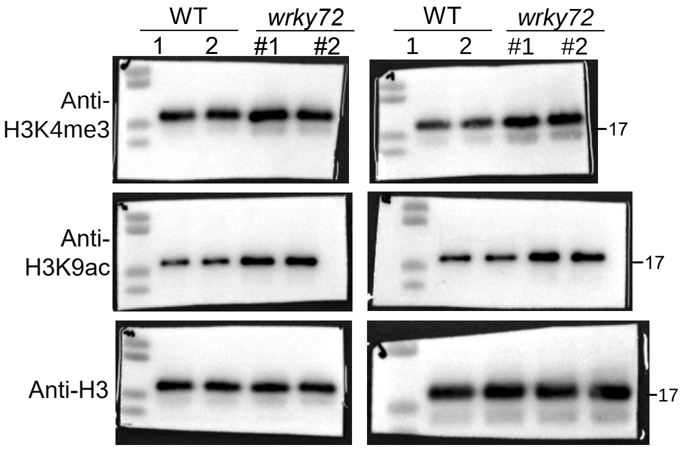

Supplement: Supplementary file 3 — Supporting File 3: advs75873‐sup‐0003‐Data.zip. [file ADVS-9999-e75873-s002.zip › Raw_Uncropped_Western_Blot_Images.pdf]
